# Photocatalytic and Electrocatalytic Properties of NGr-ZnO Hybrid Materials

**DOI:** 10.3390/nano10081473

**Published:** 2020-07-27

**Authors:** Florina Pogacean, Maria Ştefan, Dana Toloman, Adriana Popa, Cristian Leostean, Alexandru Turza, Maria Coros, Ovidiu Pana, Stela Pruneanu

**Affiliations:** National Institute for Research and Development of Isotopic and Molecular Technologies, Donat Street, no. 67-103, RO-400293 Cluj-Napoca, Romania; florina.pogacean@itim-cj.ro (F.P.); maria.stefan@itim-cj.ro (M.Ş.); dana.toloman@itim-cj.ro (D.T.); adriana.popa@itim-cj.ro (A.P.); cristian.leostean@itim-cj.ro (C.L.); alexandru.turza@itim-cj.ro (A.T.); maria.coros@itim-cj.ro (M.C.); ovidiu.pana@itim-cj.ro (O.P.)

**Keywords:** N-doped graphene-ZnO, hybrid materials, rhodamine B, N-doped graphene-modified electrodes

## Abstract

N-doped graphene-ZnO hybrid materials with different N-doped graphene:ZnO wt% ratios (1:10; 1:20; 1:30) were prepared by a simple and inexpensive sol-gel method. The materials denoted NGr-ZnO-1 (1:10), NGr-ZnO-2 (1:20), and NGr-ZnO-3 (1:30) were investigated with advanced techniques and their morpho-structural, photocatalytic, and electrocatalytic properties were reported. Hence, pure N-doped graphene sample contains flakes with the size ranging from hundreds of nanometers to micrometers. In the case of all NGr-ZnO hybrid materials, the flakes appear heavily decorated with ZnO nanoparticles, having a cauliflower-like morphology. The X-ray powder diffraction (XRD) investigation of N-doped graphene sample revealed that it was formed by a mixture of graphene oxide, few-and multi-layer graphene. After the ZnO nanoparticles were attached to graphene, major diffraction peaks corresponding to crystalline planes of ZnO were seen. The qualitative and quantitative compositions of the samples were further evidenced by X-ray photoelectron spectroscopy (XPS). In addition, UV photoelectron spectroscopy (UPS) spectra allowed the determination of the ionization energy and valence band maxima. The energy band alignment of the hybrid materials was established by combining UV–Vis with UPS results. A high photocatalytic activity of NGr-ZnO samples against rhodamine B solution was observed. The associated reactive oxygen species (ROS) generation was monitored by electron paramagnetic resonance (EPR)-spin trapping technique. In accordance with bands alignment and identification of radical species, the photocatalytic mechanism was elucidated.

## 1. Introduction

In recent years, constant efforts have been made to acquire facile and cost-effective methods to resolve environmental problems using eco-friendly and functional materials. The semiconducting materials are widely used at the degradation of organic compounds from water by photocatalytic processes. Among the semiconductors, ZnO has found application in removing various contaminants due to its nontoxic nature, low cost correlated with high photosensitivity, chemical and physical stability [1,2,3]. Despite its great qualities, ZnO has several deficiencies like the limit of UV-light adsorption due to the wide band gap, fast recombination of photo generated electron-hole pairs [4], and relatively poor electrical conductivity [5]. Therefore, it is necessary to combine it with other materials in order to improve its photocatalytic [6] and electrocatalytic activity [7].

Possessing high mobility, good conductivity, great mechanical flexibility and excellent chemical stability, graphene is a suitable modifier for improving ZnO catalytic performances. Several papers reported the use of graphene oxide (GO) [8] and its reduced form (rGO) [9] as a photocatalytic performance enhancer for ZnO [10,11]. For the synthesis of these graphene-based materials, modified Hummers method is usually employed, followed by the reduction of GO with various reducing agents [12,13]. The described synthetic procedure is complicated and leads to higher prices for the final materials. The electrochemical exfoliation of graphite is a promising way to produce high-quality graphene in an easier way and with lower costs [14,15].

Regarding the synthesis of graphene-ZnO hybrid materials, the most popular methods include sol-gel [16], hydrothermal [17], microwave [18,19], sonochemical [20], and surfactant-assisted synthesis. [21] Other methods comprise solvent free approaches as reported by Abdala et al. via short-time ball milling of hydrozincite and GO followed by thermal annealing [22] or partial combustion in muffle furnace [23]. ZnO-graphene hybrids, obtained using two different natural reducing agents, namely grape and *Eichhornia crassipes*, were used for the photocatalytic degradation of rhodamine B (RhB) dye in aqueous solution [24]. Rhodamine B is a synthetic dye used as a colorant in the manufacturing of textiles, leathers, paints, and foods. It irritates the eyes and skin being also carcinogenic and toxic for the nervous and reproductive systems. For these reasons it is necessary to remove this contaminant from wastewaters [25]. Photocatalytic degradation of such organic pollutants has gained increasing consideration as a promising technology for reducing pollution [26,27]. Although graphene-ZnO nanocomposites synthesized by various methods proved to be very successful for photocatalytic applications, it is still necessary to develop a scalable and economical route for their synthesis.

ZnO nanoparticles also exhibit attractive antimicrobial properties against bacteria (Gram-positive and Gram-negative) and fungi, due to the formation of reactive oxygen species (ROS) on their surface [28]. By combining graphene with a semiconducting material such as ZnO a synergistic effect is created that contributes to the improvement of decontamination properties [29]. The novelty of the present work is related to the simple route employed to synthesize N-doped graphene-ZnO hybrid materials, based on the electrochemical exfoliation of graphite rods followed by a sol-gel process. In order to reveal the morphological and structural characteristics of the synthesized composites, various techniques were employed, such as scanning electron microscopy (SEM) coupled with energy dispersive X-ray spectroscopy (EDS), high-resolution transmission electron microscopy (HRTEM), X-ray powder diffraction (XRD), UV–Vis spectroscopy, X-ray photoelectron spectroscopy (XPS), and UV photoelectron spectroscopy (UPS). The complex investigations by UPS allowed an accurate determination of both ionization energies and positions of the valence band maxima. UV–Vis spectroscopy was used to calculate the optical band gaps of both NGr and ZnO while electron paramagnetic resonance (EPR) coupled with the spin trapping probe technique evidenced the ROS generation process. Based on the above-mentioned experiments, the energy band alignments and subsequently the photocatalytic degradation mechanism were established. In addition, the electrocatalytic performances of glassy-carbon electrodes modified with the hybrid materials were investigated for the first time and compared with those of pure ZnO and N-doped graphene. Important electrochemical parameters, such as charge transfer resistance (R_ct_) and the apparent heterogeneous electron transfer rate constant (K_app_) for ZnO and NGr-ZnO hybrid materials are also reported for the first time.

## 2. Materials and Methods

### 2.1. Materials

Materials and reagents used for the preparation of N-doped graphene-ZnO were: N-doped graphene obtained by electrochemical exfoliation, zinc acetate (Zn(CH_3_COO)_2_·2H_2_O) (Alpha Aesar Thermo Fisher (Kandel) GmbH, Kandel, Germany), diethylene glycol (DEG), C_4_H_10_O_3_ (Sigma-Aldrich, Merck, KGaA, Darmstadt, Germany), and absolute ethanol (C_2_H_5_OH-EtOH) (Sigma-Aldrich, Merck, KGaA, Darmstadt, Germany). All chemicals were of analytical grade and used without further purification. The aqueous solutions were prepared with Milli-Q water obtained from Direct-Q 3UV system (Millipore, Bedford, MA, USA). Dimethyl sulfoxide (DMSO; >99.9%) was purchased from VWR Chemicals and 5,5-dimethyl-1-pyrroline N-oxide (DMPO; >97%), dimethylformamide (DMF), and phenol were purchased from Sigma-Aldrich, Merck, KGaA, Darmstadt, Germany.

### 2.2. Synthesis of N-Doped Graphene (NGr)

The N-doped graphene sample was obtained by exfoliation of graphite rods via pulses of current, as recently described in our paper [15]. Briefly, two graphite rods were employed as an anode and cathode in an electrochemical cell, filled with the appropriate electrolyte: 0.1 M ammonium thiocyanate (NH_4_SCN) + 0.1 M ammonia. The exfoliation took place at an applied bias of 11 V and 0.5 A current intensity (current pulse duration 0.8 s; the pause between two pulses 0.2 s; 4 h exfoliation time). At the end of the exfoliation process, the material was washed with 8 L of distilled water then dispersed by ultrasound for 30 min in 125 mL water. Next, it was filtered and finally dried by lyophilization. The obtained N-doped graphene sample was following denoted NGr.

### 2.3. Synthesis of N-Doped Graphene-ZnO Hybrid Materials (NGr-ZnO)

Hybrid materials with different NGr:ZnO wt% ratios (1:10; 1:20; 1:30) were prepared by sol-gel process, as next described. In the first stage, 1.2 mmol of Zn(CH_3_COO)_2_·2H_2_O was dissolved in a mixture of DEG:H_2_O (20:1 vol. ratio) under magnetic stirring. The obtained solution was heated at 160–180 °C for 10 min then the mixture was kept at room temperature for 3 h, to obtain the ZnO sol. In the second stage, NGr was added to the ZnO sol and sonicated for 30 min to ensure its dispersion. The decoration of NGr with ZnO nanoparticles was achieved after stirring the above mixture at 160–180 °C for 2 h. Finally, the solution was cooled down to room temperature, centrifuged and washed with deionized water and absolute ethanol then dried in the oven at 65 °C. The hybrid materials were denoted as follows: NGr-ZnO-1 (1:10 NGr:ZnO wt%), NGr-ZnO-2 (1:20 NGr:ZnO wt%), and NGr-ZnO-3 (1:30 NGr:ZnO wt%) and their morpho-structural, photocatalytic, and electrocatalytic properties were investigated.

### 2.4. Preparation of Glassy-Carbon Modified Electrodes

Five glassy-carbon (GC) electrodes were polished on a felt cloth, rinsed with double-distilled water, and dried at room temperature for several hours. From each sample dispersed in DMF (1 mg/mL) a volume of 10 µL was deposited onto the electrode surface. After modification, the electrodes were room-temperature dried for 24 h. They were correspondingly denoted as GC/NGr, GC/ZnO, GC/NGr-ZnO-1, GC/NGr-ZnO-2, and GC/NGr-ZnO-3.

### 2.5. Instruments

SEM analysis was performed using a SU8230 High-Resolution Scanning Electron Microscope (Hitachi, Tokyo, Japan) equipped with a cold field emission gun. For scanning transmission electron microscope (STEM)/EDS analysis, a drop of suspension of each sample was deposited and dried on a copper grid. The analysis was carried out using a HD-2700 scanning transmission electron microscope (STEM) (Hitachi, Tokyo, Japan), equipped with a cold field emission gun, working at an acceleration voltage of 200 kV and designed for high-resolution (HRTEM) imaging with a resolution of 0.144 nm. EDS spectra and chemical mapping for the elements were acquired using a Dual EDX System (X-Max N100TLE Silicon Drift Detector SDD) from Oxford Instruments.

Sample qualitative and quantitative compositions were investigated by using X-ray photoelectron spectroscopy (XPS) through a custom-build SPECS spectrometer (Berlin, Germany) working with monochromated Al anode (1486.71 eV). The samples dispersed in ethanol were drop-casted onto the sample holder. Quantitative analysis was performed by using CasaXPS software. A Shirley background was extracted from all spectra. The integral intensities were calibrated by the corresponding relative sensitivity (RST), transmission (T), and electronic mean free path factors (MFP) from CasaXPS data base. The integral intensities were also normalized by dividing them with the corresponding values of the escape depth. They were calculated following previous research (see [30]). They were 0.62 nm for Zn 2p and 2.65 nm for C 1s core-level kinetic energies corresponding to ZnO and N-doped graphene, respectively.

The X-ray powder diffraction (XRD) patterns of NGr, ZnO, and NGr-ZnO hybrid materials were recorded with DIFFRAC plus XRD Commander Package on a Bruker-D8 Advance Diffractometer with the tube set at 40 kV and 40 mA. A germanium (1 1 1) monochromator was placed in the incident beam (λ = 1.54056 Å) and the scan rate was 0.02 s^−1^.

The optical response of all samples was investigated by UV–Vis reflection/absorption spectroscopy. The spectra were recorded with a JASCO-V570 UV–Vis-NIR Spectrophotometer (JASCO Deutschland GmbH, Pfungstadt, Germany) equipped with JASCO ARN-475 absolute reflectivity measurement unit. The transformation of reflectance spectra in absorbance spectra was done with internal software.

The photocatalytic activity of the samples was studied by measuring the degradation rate of rhodamine B (RhB) dye solution, at room temperature under UV light illumination. The UV-irradiation was provided by two 15 W UV lamps with a peak wavelength of 365 nm. 5 mg of ZnO or NGr-ZnO hybrid samples and respective 1.25 mg of N-doped graphene were suspended in 10 mL aqueous solution of RhB (1.0 × 10^−5^ M). Next, the mixture was put into a beaker and agitated by magnetic stirring in the dark to achieve the adsorption/desorption equilibrium. The dye solution containing the sample was illuminated for 3 h, and the mixture (3.5 mL) was withdrawn for analysis every 30 min. After separating the photocatalyst from the dye solution by centrifugation, the dye concentration was determined by recording the absorption spectrum with an UV–Vis spectrophotometer (T90+; PG Instruments, Leicestershire, United Kingdom). A similar experiment was performed with a solution of phenol (10^−3^ M) in the presence of NGr-ZnO-3 sample.

To monitor the reactive oxygen species (ROS) production, electron spin resonance (ESR) coupled with the spin trapping probe technique was used. 5,5-Dimethyl-1-pyrroline N-oxide (DMPO) was used as spin trapping reagent. Each sample (10 mg) was dispersed in DMSO (1 mL) and homogenized in an ultrasound bath (30 min) before use. DMPO (0.2 M) was added to the suspension. The sample was prepared immediately before measurements and transferred into a quartz flat cell, optimized for liquids measurements. The measurements were recorded with a Bruker E-500 ELEXSYS X-band (9.52 GHz) spectrometer (Rheinstetten, Baden-Württemberg, Germany) in the same experimental conditions.

For electrochemical measurements (cyclic voltammetry (CV); electrochemical impedance spectroscopy (EIS)), a typical three-electrode cell coupled with a Potentiostat/Galvanostat Instrument (PGSTAT-302N, Metrohm-Autolab B.V., Utrecht, Netherlands) was employed. The working electrode was either bare GC or GC-modified electrode while the counter electrode was a large area Pt sheet. The CV measurements were generally run between −0.1 and +0.7 V vs. Ag/AgCl, with a scan rate of 10 mV·s^−1^. The impedance spectra were measured in potentiostatic mode over 0.1–10^6^ Hz range by using a small sinusoidal excitation signal (10 mV amplitude). The recorded EIS data were fitted using Nova 1.11 software.

## 3. Results and Discussions

### 3.1. Morphological and Structural Characterization of the Materials

**Figure 1:**
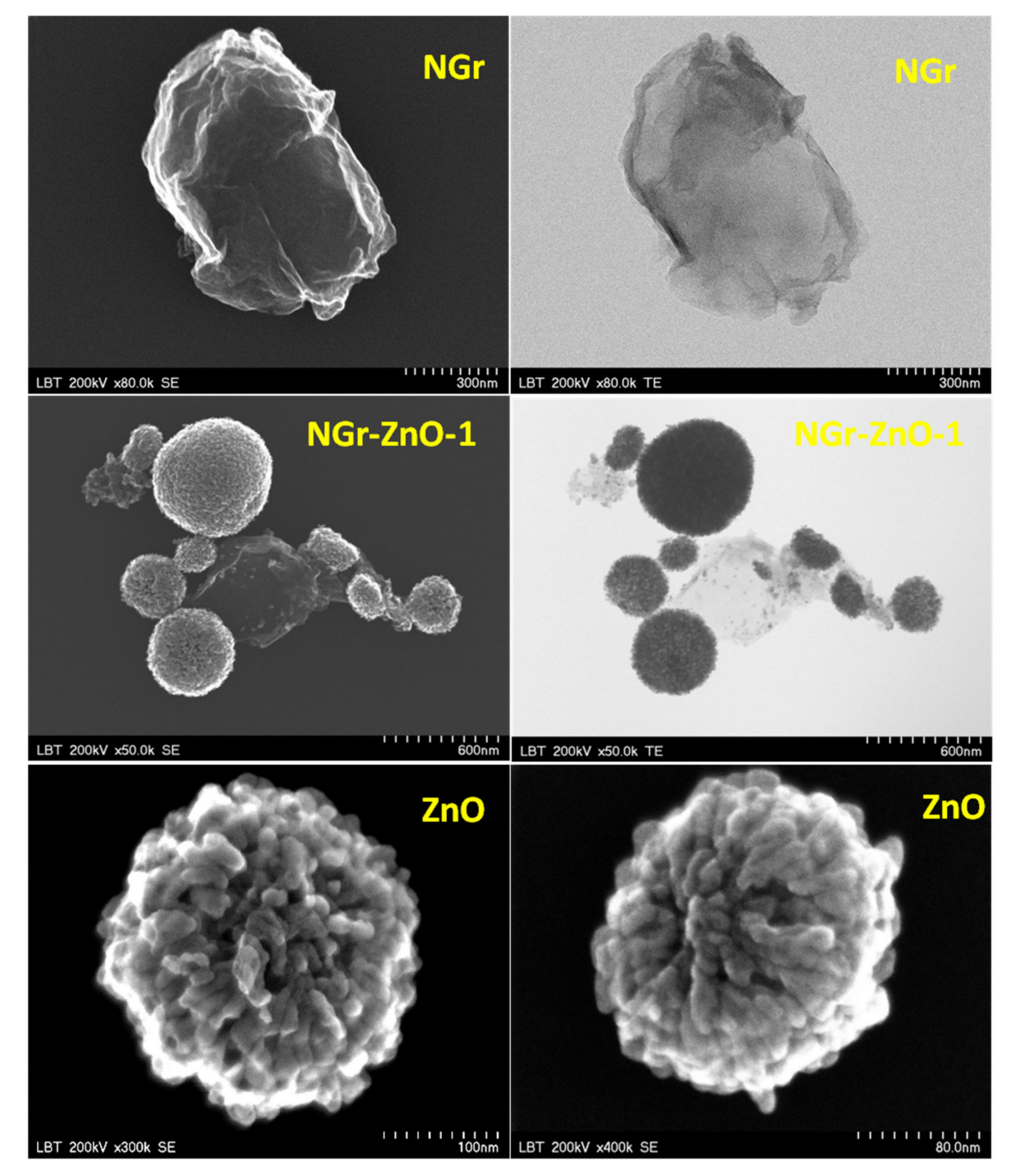
SEM and TEM image of: NGr sample (scale bar: 300 nm); NGr-ZnO-1 sample (scale bar: 600 nm); high-resolution transmission electron microscopy (HRTEM) images recorded in scanning mode for ZnO nanoparticles (scale bar: 100 and 80 nm). It can be seen that ZnO nanoparticles are grouped in bundles.

The morphological characterization of NGr, ZnO, and NGr-ZnO hybrid materials was performed using SEM/TEM techniques (Figure 1). The NGr material is formed by large flakes with the size in the range of hundreds of nanometers. In all NGr-ZnO hybrid materials, the graphene flakes appear heavily decorated with ZnO nanoparticles (see representative images for NGr-ZnO-1 sample). As it can be seen in the HRTEM image recorded in the scanning mode (Figure 1, bottom) the ZnO nanoparticles (10–20 nm) are grouped in bundles, with cauliflower-like morphology and sizes varying from 100 to 500 nm.

**Figure 2:**
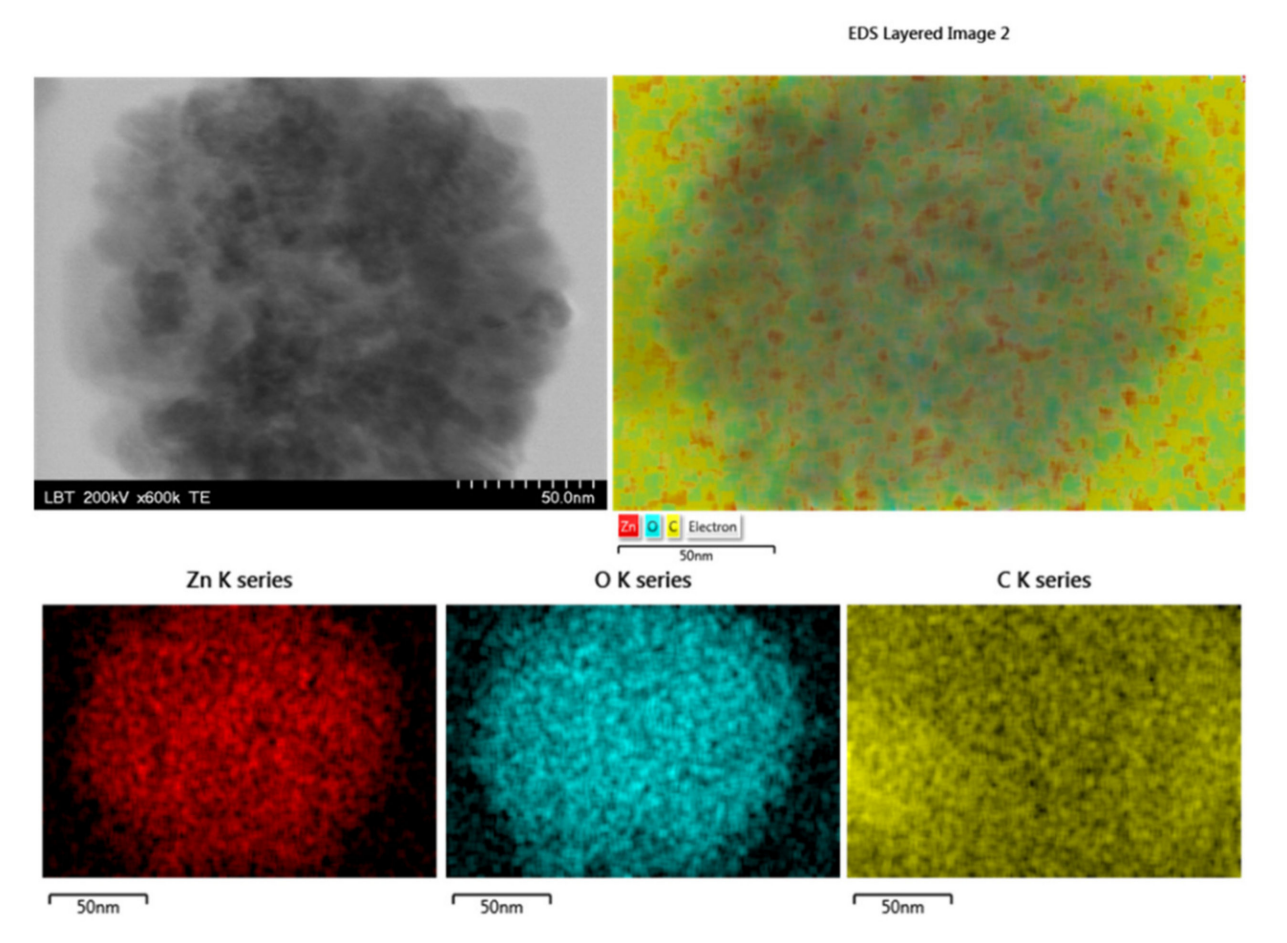
TEM image of a ZnO nanoparticle attached to graphene (NGr-ZnO-1 sample) and the corresponding energy dispersive X-ray spectroscopy (EDS) mapping, indicating the elements: Zn, O, and C.

TEM investigation coupled with elemental mapping (Figure 2) was used to display the elemental distributions of Zn, O, and C in the obtained materials (e.g., NGr-ZnO-1 sample). As can be seen in this figure, carbon is uniformly distributed within the material, while zinc and oxygen are found in ZnO nanoparticles, proving their attachment to NGr surface. 

In order to evaluate the structural changes that occurred after N-doped graphene was decorated with ZnO nanoparticles, the samples were next assessed by various characterization techniques (XRD, UV–Vis, XPS, and UPS).

**Figure 3:**
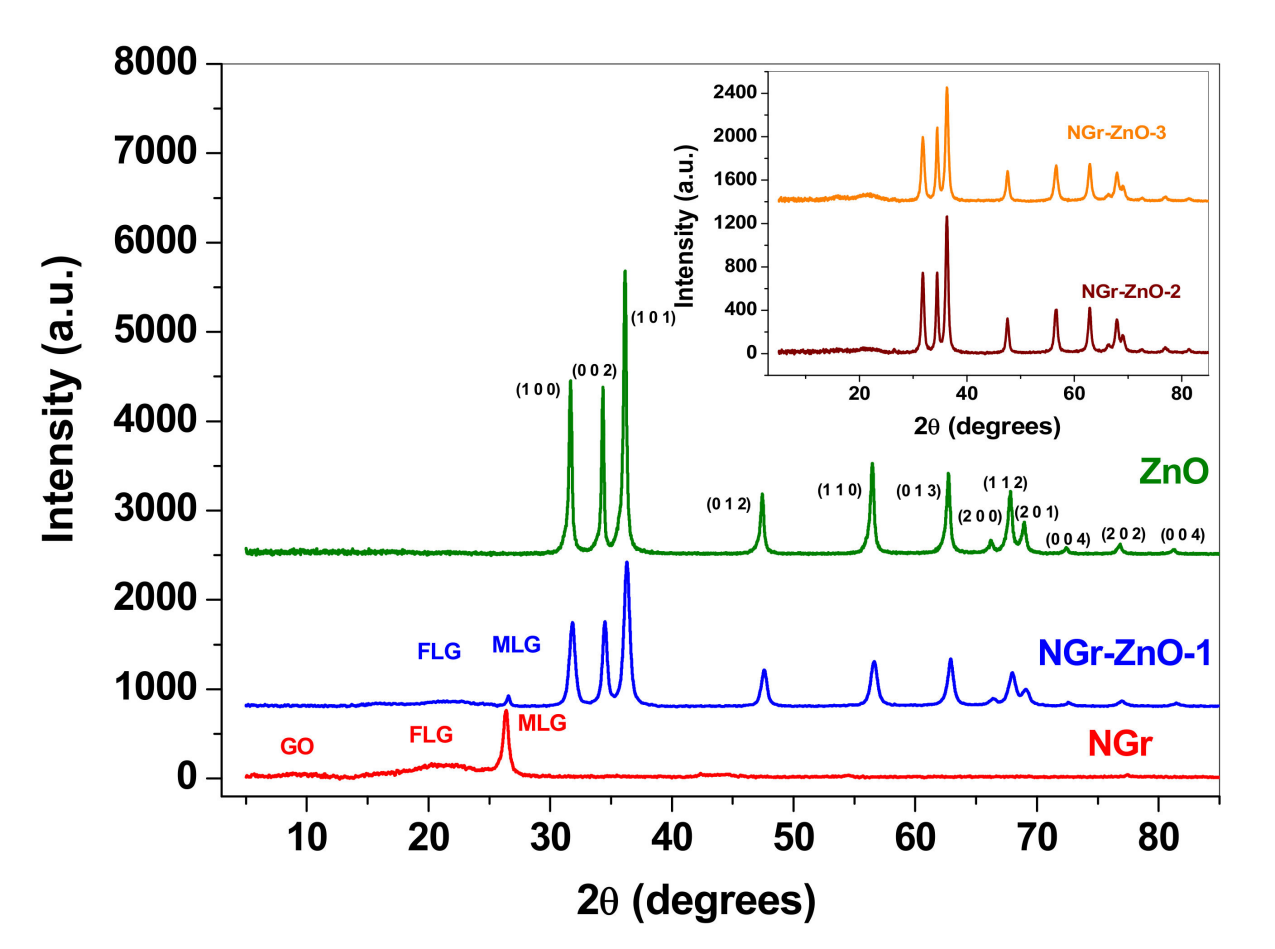
X-ray powder diffraction (XRD) pattern of: NGr, NGr-ZnO-1, and ZnO; inset: the patterns of NGr-ZnO-2 and NGr-ZnO-3 samples.

Figure 3 shows the XRD patterns of NGr and NGr-ZnO-1 samples and compares with the pattern of raw ZnO. Major diffraction peaks of ZnO can be seen at 2θ values of 31.8°, 34.4°, 36.2°, 47.5°, 56.5°, 62.7°, 66.3°, 68.0°, 69.1°, and 76.9° which correspond to (100), (002), (101), (012) (110), (013), (200), (112), (201), and (202) crystalline planes. These crystalline planes are indexed to the zincite structure of ZnO (JCPDS card no. 80-0075) [31].

The XRD pattern of the NGr sample indicates that it consists of a mixture of graphene oxide (GO; 2θ = 9.2°), few-layer graphene (FLG; 2θ = 21.84°), and multi-layer graphene (MLG; 2θ = 26.36°). After the ZnO nanoparticles were attached to N-doped graphene (e.g., NGr-ZnO-1 sample), the characteristic peaks of graphene were considerably reduced. This can be attributed to the good crystallinity of ZnO nanoparticles which weakened the diffraction of carbon atoms in graphene. Similar patterns were obtained for NGr-ZnO-2 and NGr-ZnO-3 samples, as shown in the inset of Figure 3. 

The mean crystallite size (<*D*>) and the strain (*ε*) for the zinc oxide phase (Table 1) were evaluated by Williamson–Hall plot, using Powder Cell 2.3 software [32]. The raw ZnO material is characterized by large crystallites (348 Å), which decreased in size after mixing with graphene, in the order ZnO > NGr-ZnO-1 > NGr-ZnO-2 > NGr-ZnO-3. The mean size determined from the analysis of the XRD patterns is in accordance with the HRTEM investigation. As expected, the strain also shows a small variation and increases with the decrease of the crystallite size (see Table 1).

**Figure 4:**
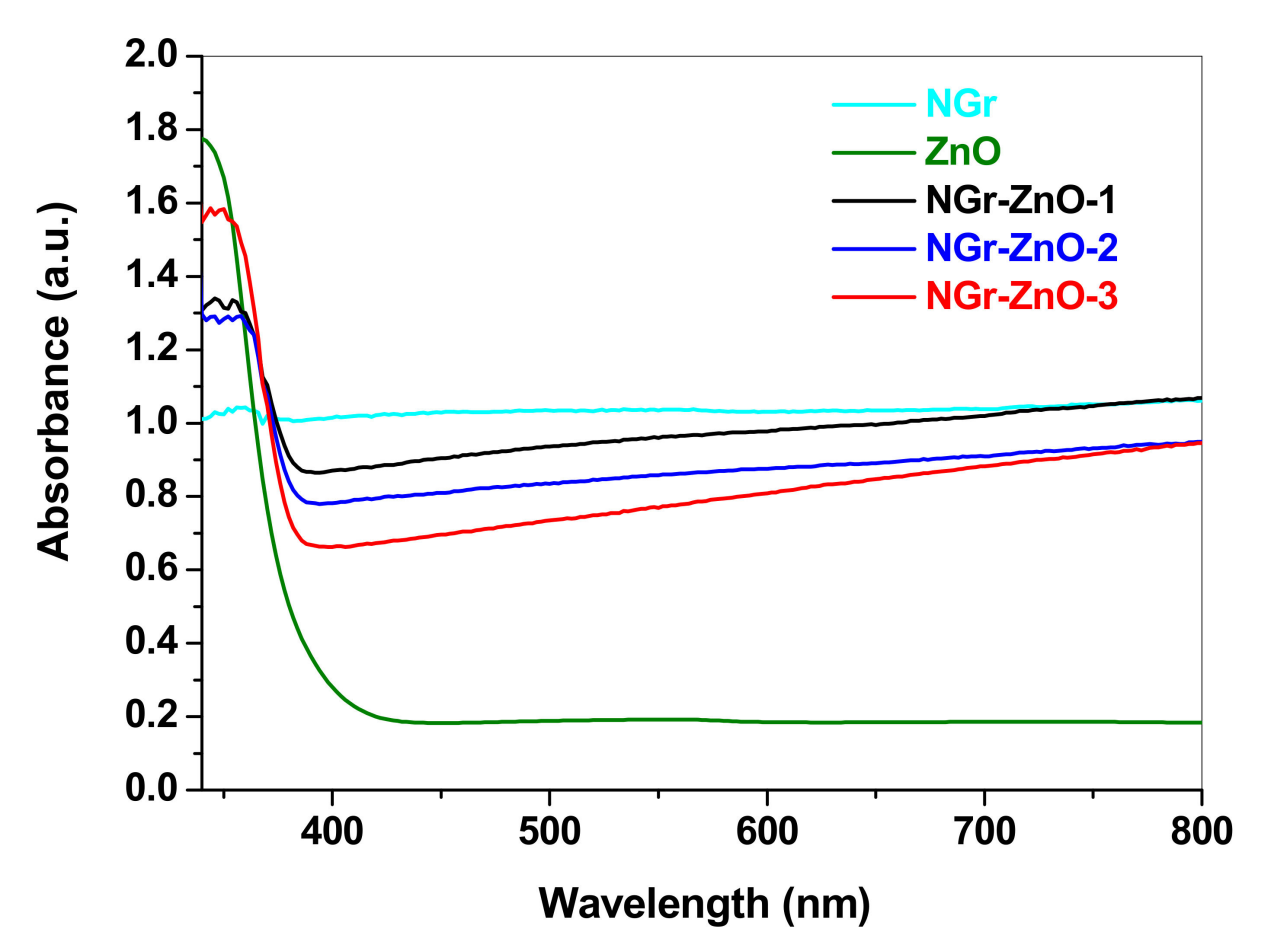
UV–Vis absorption spectra of NGr, ZnO, and NGr-ZnO hybrid samples.

The optical properties of the synthesized materials were studied by UV–Vis spectroscopy. The room temperature UV–Vis absorption spectra of NGr, ZnO, and NGr-ZnO hybrid samples were examined over the 200–800 nm range and shown in Figure 4. All samples exhibit strong absorption bands in the ultraviolet region, between 335 and 355 nm. In the case of ZnO and NGr-ZnO hybrids, the strong light absorption at around 350 nm is the result of the intrinsic band gap of ZnO. The blue shift of the absorption band in ZnO nanoparticles compared to bulk ZnO (380 nm) is characteristic of nano-sized ZnO due to quantum confinement [33]. One can observe that for NGr-ZnO samples the absorption edge shifts toward a longer wavelength (red shift) indicating that the addition of graphene to ZnO modifies the spectral response to visible light absorption. 

**Figure 5:**
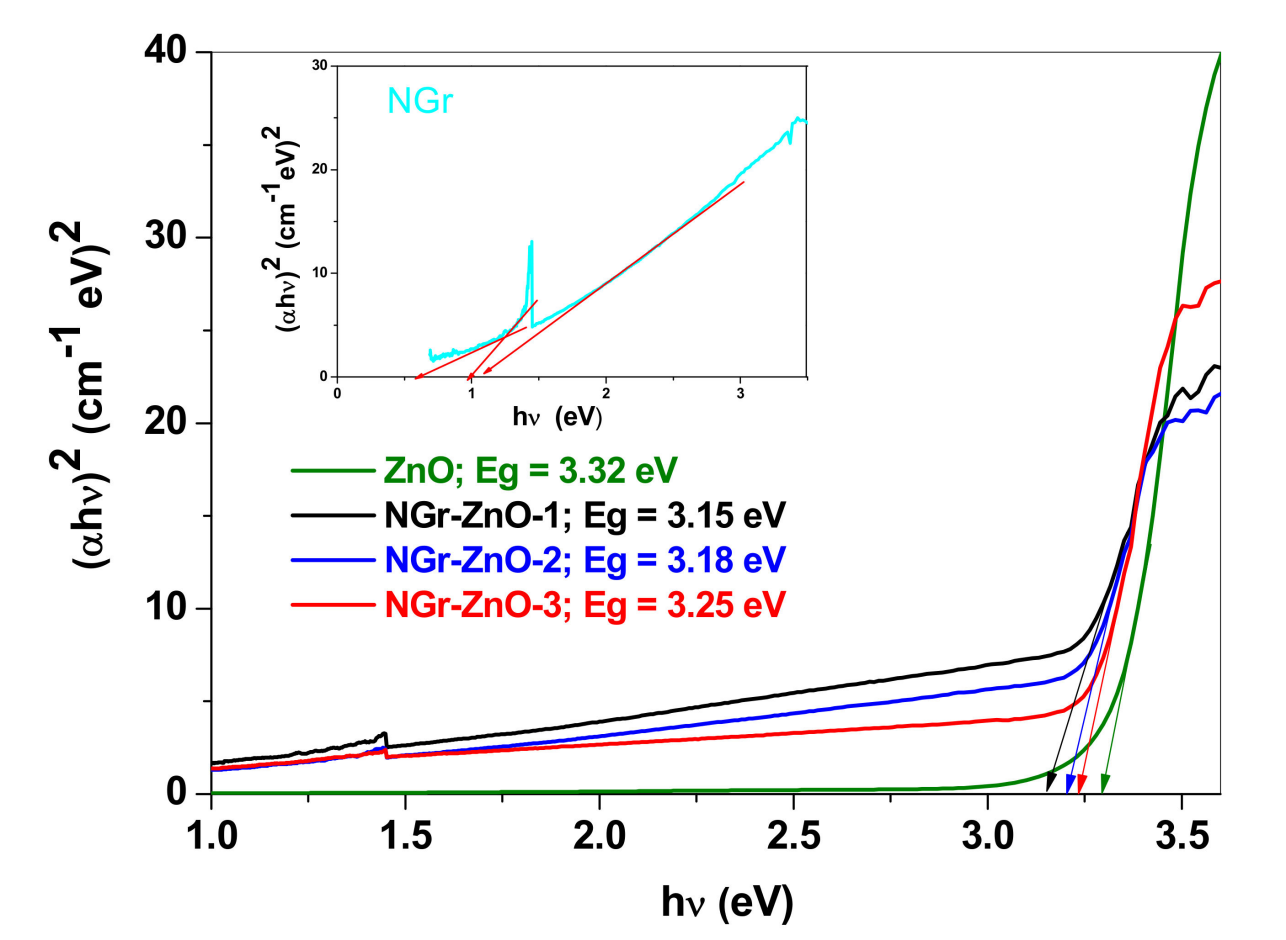
Tauc plots for ZnO and NGr-ZnO hybrid samples; inset: Tauc plot for NGr.

Considering that ZnO is a direct band gap semiconductor, the value of energy band gap (*E_g_*) was calculated from the UV–Vis absorption data using the Tauc equation: (*αhν*)^2^ = *A*(*hν* − *E_g_*), where *hν* is the photon energy and *α* is the optical absorption coefficient. A linear dependence is obtained by plotting (*αhν*)^2^ = *f*(*hν*), which indicates the type of predominant transition as directly allowed for all investigated samples. By extrapolating the linear region of the curve to zero absorption, as shown in Figure 5, the band gap values of ZnO and NGr-ZnO samples were determined. From the Tauc plot it can be seen that at low photon energies (1–3 eV) the ZnO absorption approaches zero, so the nanoparticles are transparent to visible radiation. After mixing with N-doped graphene in various ratios, the absorbance considerably increases, and the absorption edge shifts towards lower energy. At energies above 3.4 eV the absorption process saturates, and the curves deviate from linearity.

The band gap values for the investigated samples were found to be: 3.15 eV (NGr-ZnO-1), 3.18 eV (NGr-ZnO-2), and 3.25 eV (NGr-ZnO-3), being lower than that of pure ZnO (3.32 eV) [34]. The inset in Figure 5 shows that for NGr sample, several variants for the forbidden bandwidth are possible. NGr forms a statistical ensemble of flakes with different degrees of nitrogen doping as well as with different amounts of sheets in a given flake. Through the insertion of energy levels inside the NGr band gap [35], as is the case of N-doping into the pyrrolic and pyridinic positions, the width of the forbidden band decreases with the increase of nitrogen doping at these non-graphitic positions. The doping into substitution positions (graphitic nitrogen) acts in reversed sense, causing the increase of the band gap. The band gap corresponding to N-doped graphene within the hybrid materials was calculated using the extended linear portions of Figure 5 (energies between 1.5 and 3 eV). 

We suggest that the flakes which have a high degree of non-substitution level of nitrogen doping are mainly selected during the formation process of NGr-ZnO. This is favored by the acidity (~pH 4) of the reaction solution. The band gap calculated for NGr (within NGr-ZnO hybrid materials) has different values compared to the stand-alone NGr sample. As a reference for the stand-alone NGr we considered the lowest value of the energy gap (~0.5 eV) corresponding to the maximum pyridinic and pyrrolic nitrogen doping positions.

**Figure 6:**
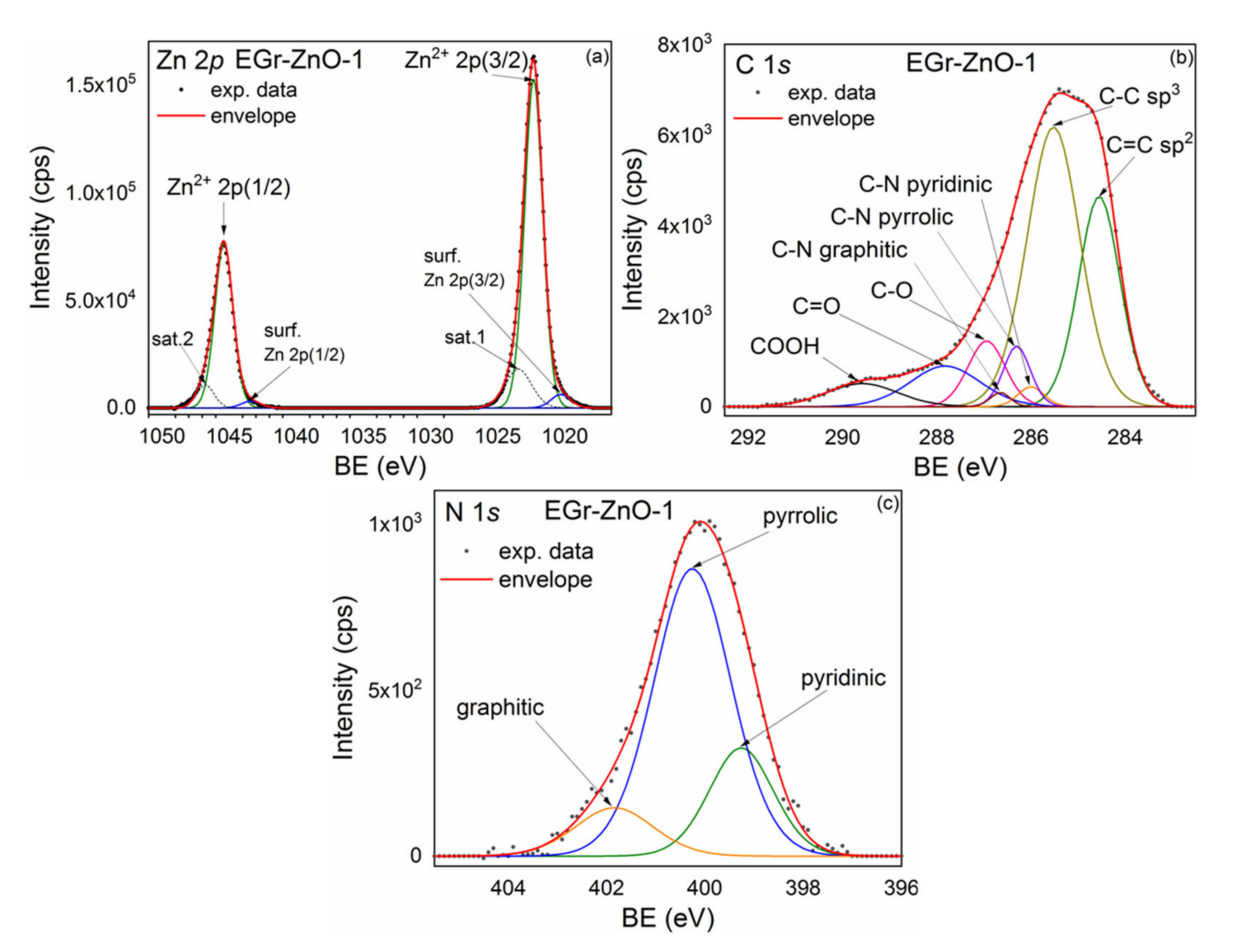
X-ray photoelectron spectroscopy (XPS) spectra corresponding to NGr-ZnO-1 sample: Zn 2p (**a**); C 1s (**b**); N 1s (**c**).

Samples qualitative and quantitative compositions were investigated by X-ray photoelectron spectroscopy (XPS). As an example, the XPS spectrum of Zn 2p core-level recorded for NGr-ZnO-1 is presented in Figure 6a. Here, the high-intensity doublet corresponds to the lattice Zn atoms in ZnO, while the low-intensity doublet, positioned at lower binding energies, represents the Zn atoms from the surface of the ZnO nanocrystals. The deconvolution was performed taking into account a spin–orbit splitting (doublet separation) of 23.07 eV. The peak areas were set as A_(2p-1/2)_ = (1/2) A_(2p-3/2)_. Two small shake-up satellite features are also seen at higher binding energies.

To account for the NGr in the hybrid samples, the C 1s core-level line was also acquired and is displayed in Figure 6b. The deconvolution was done by taking into account the specific lines for the graphene, C=C sp^2^, C–C sp^3^, C–O/C–OH, C=O, and COOH [36]. The binding energy positions are 284.5 eV for C=C sp^2^, 285.5 eV for C–C sp^3^, 286.0 eV for C–N pyridinic, 286.3 eV for C–N pyrrolic, 286.6 eV for C–N graphitic, 286.9 eV for C–O, 287.8eV for C=O, and 289.6 eV for COOH. The presence of ZnO nanoparticles induces defects (sp^3^) within the graphene structure. It was observed that going from NGr-ZnO-1 to NGr-ZnO-3 sample the sp^3^/sp^2^ ratio increases, so the number of structural defects increases with the ZnO content. The peaks of oxygen-containing groups C–O, C=O, and COOH also increase with the increase of ZnO content. The C–N component was correlated with N 1s deconvolution presented in Figure 6c. The pyridinic, pyrrolic, and graphitic peaks were set to the same normalized areas as for N 1s core levels [37].

The N 1s core level lines spectrum corresponding to NGr-ZnO-1 sample is presented in Figure 6c. The deconvolution was done by taking into account the specific pyridinic, pyrrolic, and quaternary lines for the graphene. The intense pyrrolic line is positioned at 400.2 eV while the smaller pyridinic and graphitic lines are positioned at 399.2 and 401.8 eV, respectively. By using the deconvoluted peaks’ integral intensities, the total N concentration in the hybrid samples as well as the No_(Z)_/No_(C-Gr)_ atomic ratios were determined (No_(z)_ is the number of atoms from Z species). An observation should be done here: the nitrogen concentration is higher within the hybrid samples than in the stand-alone N-doped graphene. As indicated by EDS analysis, not all the graphene flakes contain nitrogen. It sustains the above assumption that the nucleation of ZnO nanocrystals in the graphene suspension is selective such that those graphene flakes which contain more nitrogen contribute to the formation of the hybrids. When the ratio between NGr and ZnO decreases, this type of selectivity increases, thus a much higher concentration of nitrogen resulted for NGr-ZnO-3 sample. 

Taking into account the preparation method of NGr, we also looked for sulfur content inside the samples. It was detected only in the case of the bare NGr sample. The deconvolution process indicates two components, namely S from thiol, positioned at 163.7 eV BE, and S from [SCN]^−^ ion at 168.1 eV BE. Its atomic content, with respect to total nitrogen, has the following ratios: S^thiol^/N = 0.03 and S^[SCN]−^/N = 0.1. It appears that the [SCN]^−^ ions either did not associate with the zinc in solution or were decomposed by the final thermal treatment and, as a result, the sulfur does not appear in the hybrid materials or is below the detection limit.

### 3.2. Photocatalytic Studies

The photocatalytic activity of the samples against RhB synthetic solution was evaluated under UV irradiation. Before UV irradiation, the samples were kept in RhB synthetic solution in the dark, to achieve adsorption equilibrium (1 h). The adsorption of N-doped graphene sample is about 85%. The high adsorption capacity is based on the strong interactions between the surface functional groups of N-doped graphene and the active functional groups of RhB that in general overcome the weaker electrostatic forces between water/N-doped graphene/dye. The in-situ growth of ZnO nanoparticles leads to a decrease in the adsorption capacity of hybrid samples which varies between 36% for NGr-ZnO-1 and 23–26% for the other two samples (Table 2). The decrease in the adsorption capacity may be due to the ZnO nanoparticles binding the OH functional groups of the graphene surface. 

**Figure 7:**
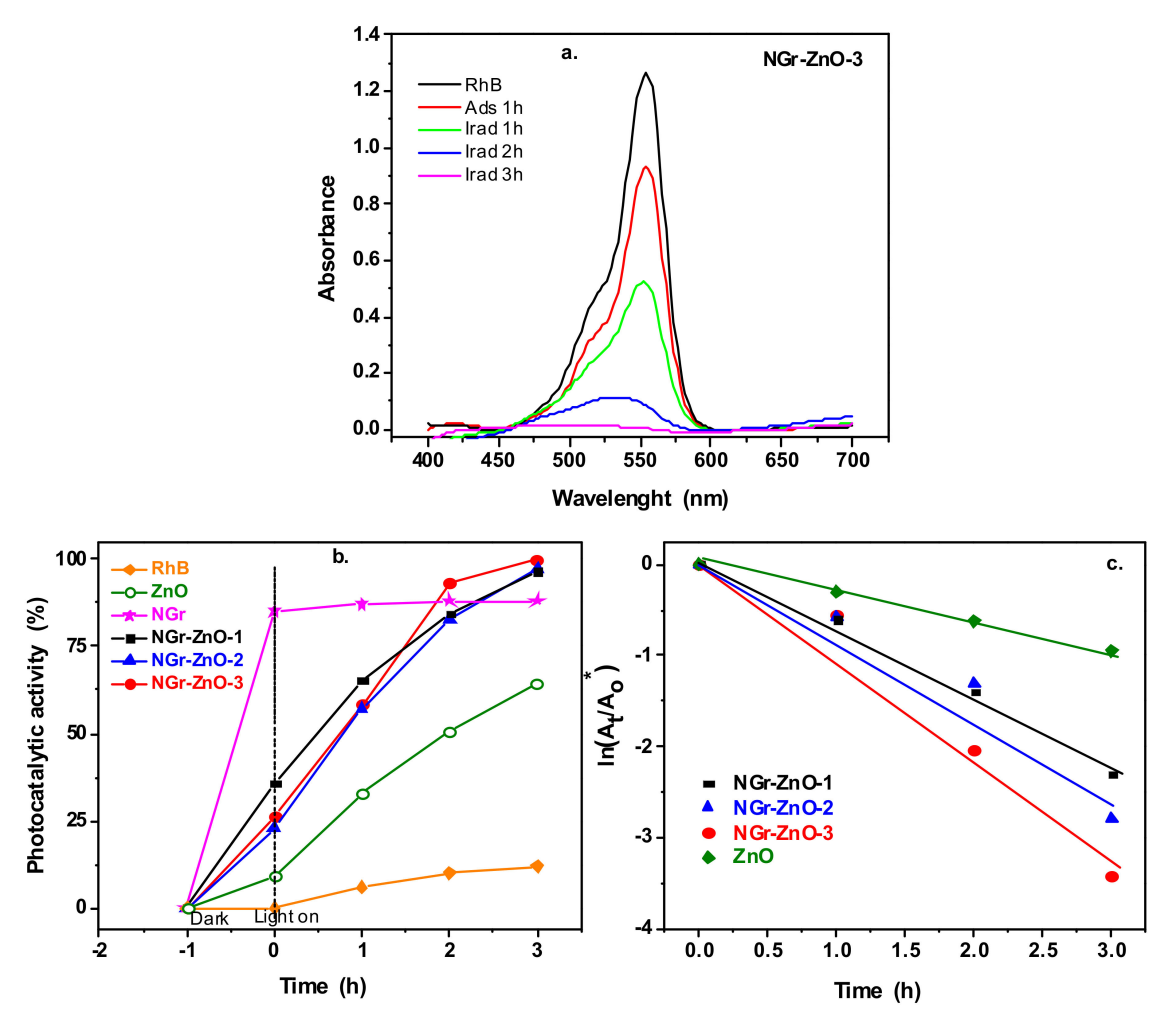
The absorbance of rhodamine B (RhB) solution in the presence of NGr-ZnO-3 sample after 1 h dark adsorption and at different irradiation times (**a**); the photocatalytic activity of the samples (**b**); evaluation of the photodegradation kinetic (**c**).

The photocatalytic activity was evaluated by measuring the absorbance of the specific band of RhB (554 nm) at different irradiation times. As an example, Figure 7a shows the absorbance of RhB in the presence of NGr-ZnO-3 sample.

The photocatalytic activity of RhB solution under UV light irradiation and in the presence of the prepared materials is presented in Figure 7b. This figure also shows the photolysis of the RhB solution, without photocatalyst. As can be seen, no considerable self-degradation was obtained. Among all samples, NGr-ZnO-3 had the highest photocatalytic activity, with RhB degradation (100%) achieved after 3 h of irradiation. All hybrid samples have an increased photocatalytic activity compared with ZnO nanoparticles. No photocatalytic activity was observed for pure N-doped graphene sample (NGr). 

The photocatalytic activity was calculated with the following equation:

(1) $$\mathrm{Photocatalytic}\;\mathrm{activity}\;\left(\%\right)=\frac{A_{0}-A_{t}}{A_{0}}\times 100$$

where *A_o_* and *A_t_* represent the initial and the absorbance of RhB at time *t*, respectively (at 554 nm wavelength).

The first-order kinetic model was used to describe the photocatalytic process (Equation (2)):

(2) $$-ln\left(\frac{A_{t}}{A_{0}^{*}}\right)=k_{i}t$$

where *A_t_* represents the absorbance of RhB at time *t*, $A_{0}^{*}$ the absorbance of RhB after dark adsorption, and *k_i_* the apparent kinetic constant.

The obtained plots show a linear relationship with the irradiation time (Figure 7c). The *k_i_* values obtained after the linear fitting of the curves are presented in Table 2. These values indicate that the best photocatalytic activity was obtained using NGr-ZnO-3 sample as photocatalyst.

**Figure 8:**
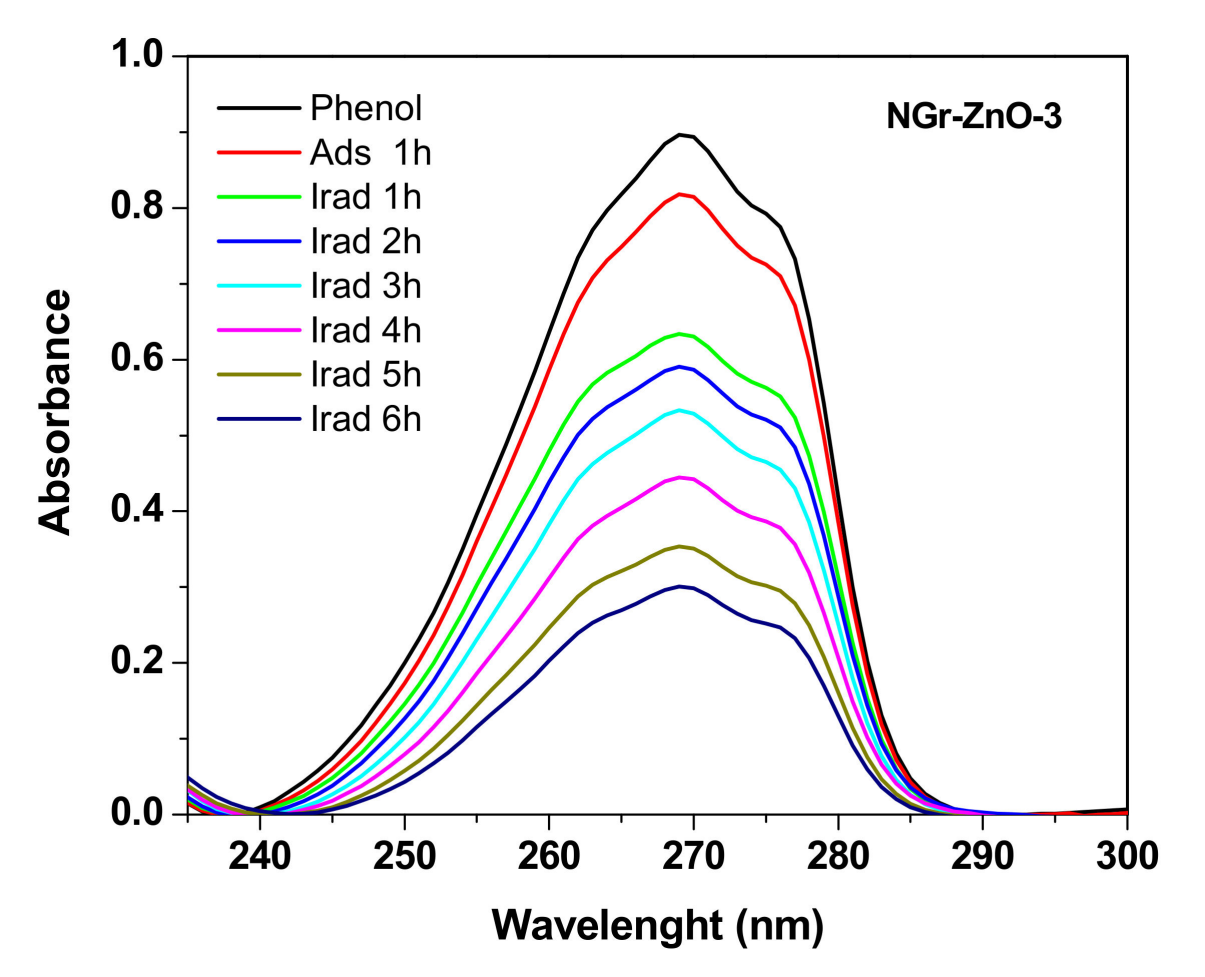
The absorbance of phenol solution in the presence of NGr-ZnO-3 sample after 1 h dark adsorption and at different irradiation times.

To eliminate the doubt of photosensitization, the NGr-ZnO-3 sample was also tested to evaluate its performance regarding phenol degradation, a colorless pollutant. The evaluation of the adsorption capacity and the photocatalytic activity was done by monitoring the UV–Vis absorbance of the specific band of phenol, centered at 269 nm. The adsorption–desorption equilibrium was reached in 1 h, with the sample presenting an adsorption rate of about 9%. As seen in Figure 8, the intensity of the specific band of phenol decreases with the increase of the irradiation time. The photocatalytic activity reaches 67% after 6 h of UV light irradiation.

A comparison between our results and previously reported works of UV or visible light induced photocatalytic degradation of different organic pollutants is presented in Table 3. Taking into account that in our work we used a lower photocatalyst concentration, the obtained photocatalytic activity is improved.

The photocatalytic activity may be explained based on the following mechanism: under UV light irradiation, electron-hole pairs are generated in the ZnO crystallites. If they do not recombine, they will migrate to the crystallite surface. Based on the literature papers, an interfacial charge transfer process between ZnO and N-doped graphene takes place [34,44,45]. More precisely, by UV irradiation the photogenerated electrons and holes from ZnO will interact with H_2_O and O_2_, generating reactive oxygen species (ROS): ·OH and ·$O_{2}^{-}$.

**Figure 9:**
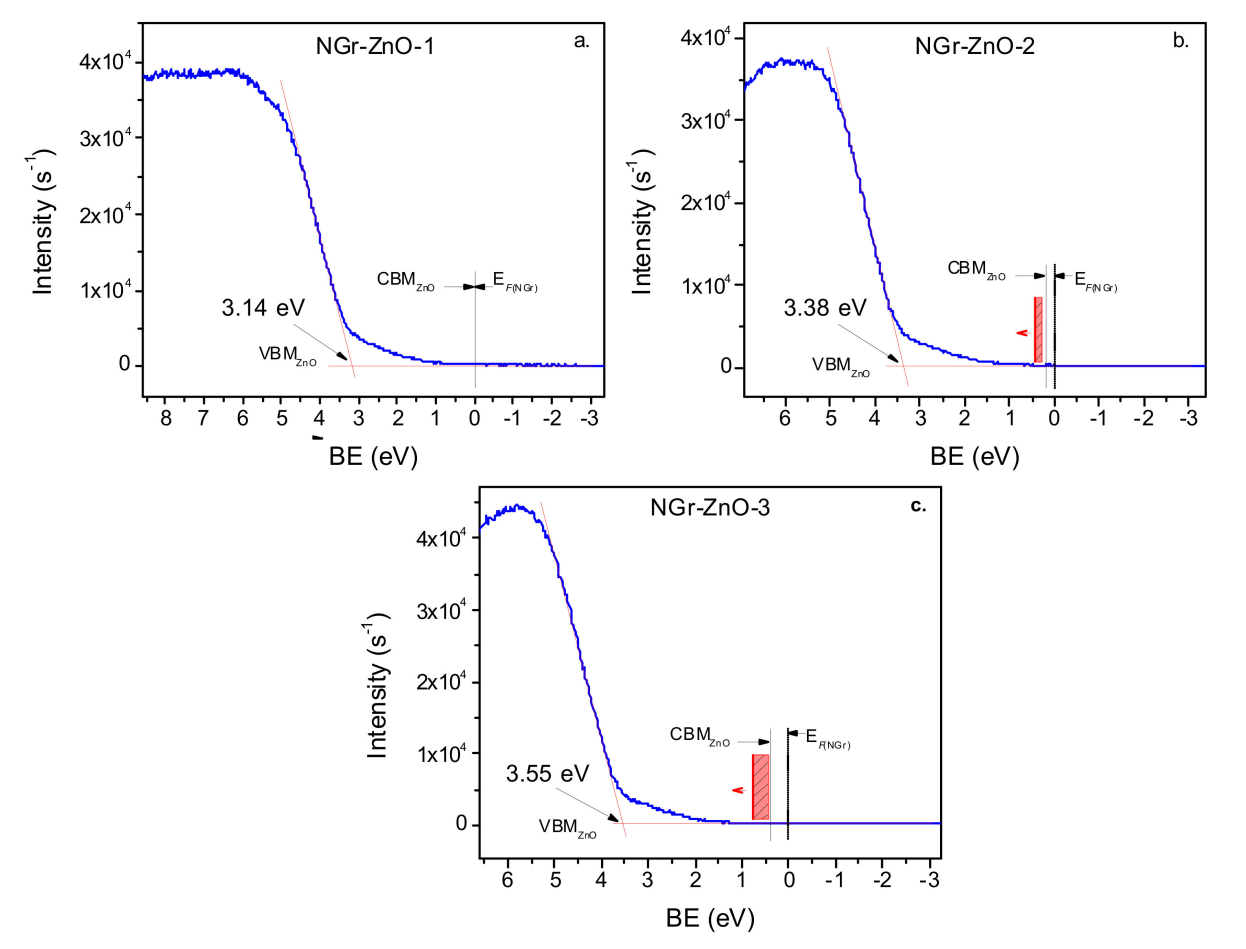
UPS spectrum of NGr-ZnO-1 (**a**), NGr-ZnO-2 (**b**), and NGr-ZnO-3 sample (**c**); The Burstein–Moss effect is shown schematically by the red rectangles and its orientation by the attached arrows. In the case of the NGr-ZnO-1 sample the effect is quite small and has not been outlined in the graph.

To further understand the ROS generation process, it was necessary to determine the relative alignment of the energy bands for the NGr and ZnO as accurately as possible. For that purpose, we used the UPS measurements which in combination with the band gap value determined from optical measurements, allowed the calculation of the ionization energy, the valence band maximum (VBM) position, conduction band minimum (CBM) position as well as the position of the Fermi level, all with respect to the vacuum energy. The ionization energy and the position of the valence band maximum may be determined directly from the UPS spectra of the hybrid materials. These spectra, in the regions of the VBM, are seen in Figure 9a–c. The values of the ionization potentials were determined from the onset of the secondary electrons emission binding energy subtracted from He–I excitation energy (21.22 eV). The positions of the VBM were obtained by extrapolating to zero the valence band (VB) slopes of the density of states. They are marked by arrows in Figure 9 (see also Table 4).

**Figure 10:**
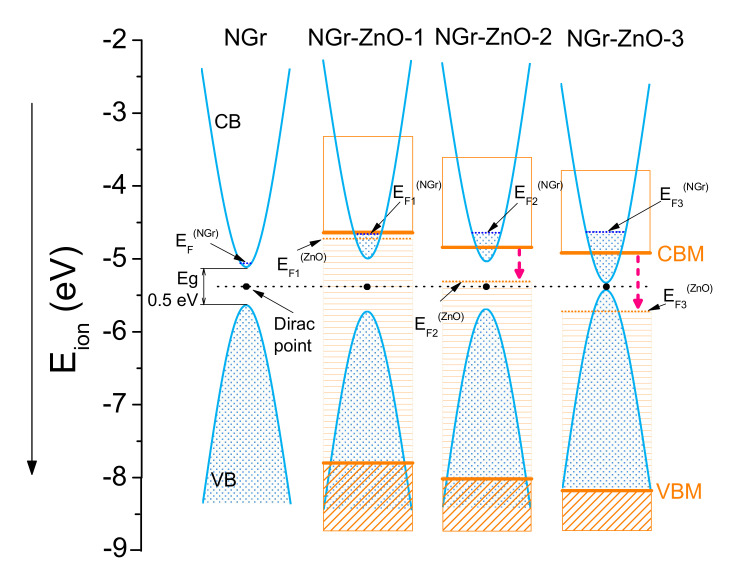
Alignment of energy bands for NGr and for the three hybrid samples. The energies are scaled with respect to vacuum energy. At the same time, the inverse Burstein–Moss effect produces a downward shift of the Fermi level and ZnO energy bands. In the case of the last two samples, the process is indicated by red dashed arrows.

By combining the results obtained from the UPS with the forbidden energy gap determined by optical spectroscopy (UV–Vis), the energy band diagram of the hybrid materials was constructed and presented in Figure 10. The presence of nitrogen in graphene causes an additional “*n*” type doping so inside the ZnO crystallites, electron depletion occurs (“*p*” doping). In the latter case, the “*p*” type doping is accompanied by an inverse Burstein–Moss (B-M) effect which produces a downward shift of the Fermi levels. In Figure 10, the levels located in the forbidden band of ZnO are represented by horizontal lines (orange). The localized level with the highest energy represents the Fermi energy of ZnO in the corresponding material. In the case of the NGr-ZnO-1 sample, the B-M effect is quite small, and the levels located in the band gap practically reach the CBM.

From the analysis of Figure 9 and Figure 10, several conclusions can be drawn: (i) Through the formation of ZnO-N-doped graphene hybrids at the interface between the two components, an electron transfer process takes place from the metal atoms to graphene; this process causes an additional “*n*” type doping of the graphene and an electron depletion (“*p*” type doping) in ZnO nanocrystallites. This depletion goes from the surface of the ZnO crystallites inwards. (ii) As a consequence, the Fermi levels do not equalize at the interface between the two components and a potential barrier, which maintains this difference between the Fermi levels, appears here. (iii) The additional doping due to electrons transferred from ZnO into N-doped graphene leads to a narrowing of the latter band gap; the most probable mechanism for this additional transfer is the deprotonation of amine or carboxamide groups of doped graphene flakes followed by their attachment to Zn atom surface. (iv) At the same time, the forbidden energy band of ZnO slightly increases and, as a result of the “*p*” type doping, an inverse Burstein–Moss effect is produced; therefore, the Fermi level of ZnO is pushed down towards the valence band simultaneously with the lowering of both CBM and VBM positions.

Following these observations, the mechanism of photocatalysis in these hybrids materials can be established: by the UV excitation at ~3.4 eV (364 nm) the holes generated in the VB of ZnO will move onto the localized and partially occupied states, positioned inside the band gap, from where they can finally reach the high mobility top of the NGr valence band; from here the holes will react with OH^−^ radicals and will generate ·OH ROS. On the other hand, the excited electrons in the conduction band (CB) of ZnO may or may not jump into the NGr conduction band. In the case of NGr-ZnO-1 and NGr-ZnO-2 samples, direct electron transitions into the ZnO CB takes place with the conservation of the wave vectors, and so the final excited states lies above the Fermi level of NGr. From these positions the excited electrons can reach the Fermi level of NGr causing the increase of the recombination probability with holes which are already present inside the NGr VB. 

**Scheme 1:**
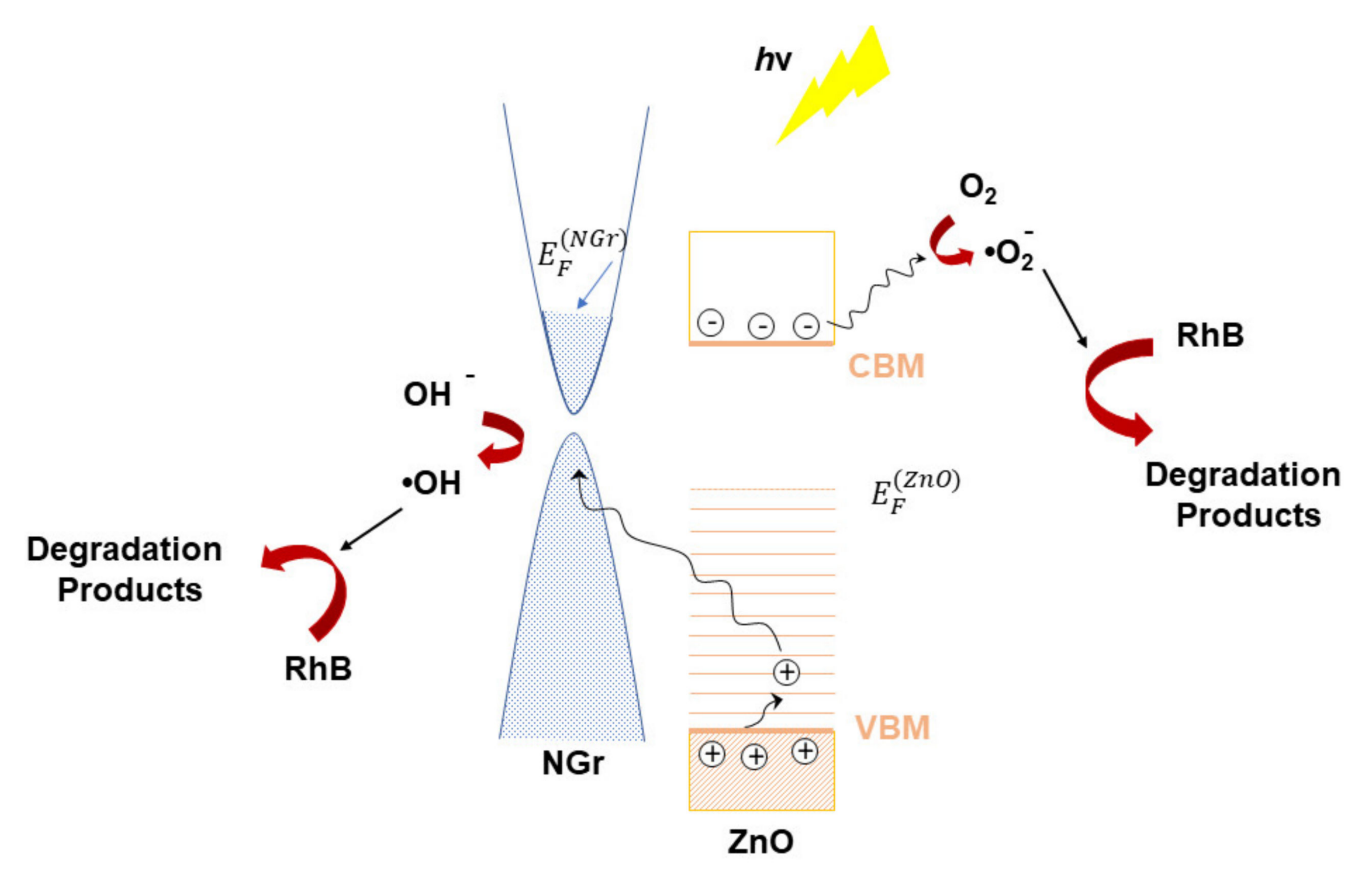
Schematic representation of photocatalytic mechanism for NGr-ZnO-3 sample.

For NGr-ZnO-3 sample, due to the larger inverse B-M effect, the final energy of the excited electrons is below the Fermi level of NGr, thus they cannot enter the NGr CB. As a consequence, their lifetime is increased and most of these excited electrons will move to the ZnO nanocrystallite surface and generate ·$O_{2}^{-}$ reactive oxygen. In other words, the ·OH species are generated mostly at the N-doped graphene–liquid interface, while ·$O_{2}^{-}$ appears on the surface of ZnO nanocrystallites. A schematic representation of the photocatalytic mechanism specific to NGr-ZnO-3 sample is presented in Scheme 1.

**Figure 11:**
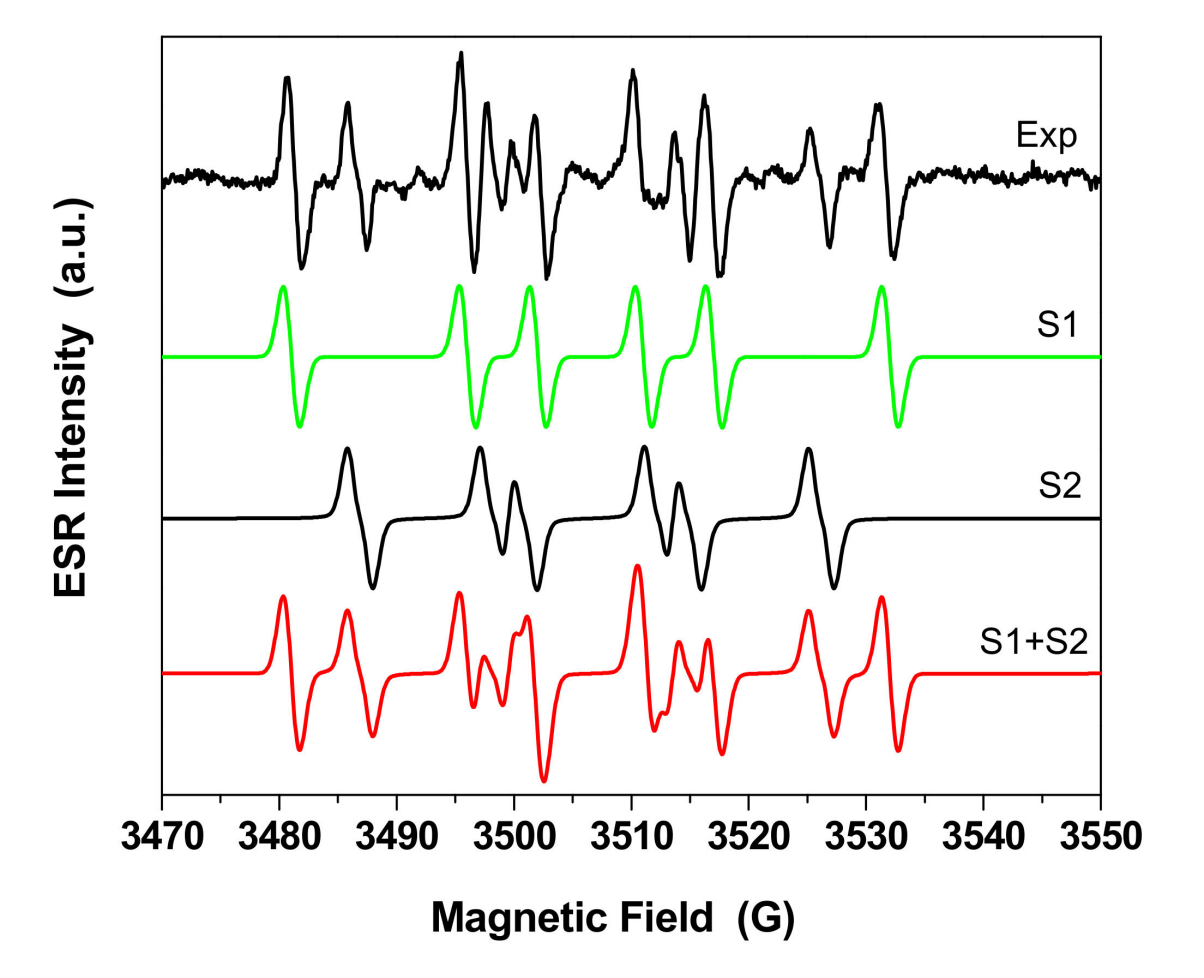
Experimental and simulated spectra of 5,5-dimethyl-1-pyrroline N-oxide (DMPO) spin adducts generated by NGr-ZnO-3 sample after 5 min of irradiation.

The ROS species interact with pollutant molecules adsorbed on the composite surface. These species are short life radicals and they are evidenced using electron spin resonance (ESR) experiment coupled with spin-trapping technique. For this method, DMPO was used as spin trapping agent and the samples were dispersed in DMSO. The ESR spectrum of DMPO spin adducts generated by NGr-ZnO-3 sample after 5 min of irradiation is presented in Figure 11.

A multiline resonance ESR spectrum was obtained due to different spin adducts generated in the irradiated system. To elucidate the spin adducts, a simulation of the spectrum was done as seen in Figure 11. The experimental spectrum could be simulated by linear combination of the following spin adducts: ·DMPO-CH_3_ (S1: a_N_ = 15 G, a_H_ = 21 G, g = 2.0090, dH = 1.4 G, relative concentration 55%) and ·DMPO-OOH (S2: a_N_ = 14 G, a_H_ = 11.3, a_H_ = 1.1 G, g = 2.0095, dH = 1.25, relative concentration 45%). The presence of ·DMPO–CH_3_ confirms the presence of ·OH radical in the system, since these spin adducts are generated by reaction between ·OH radicals and DMSO solvent [46,47]. ·DMPO-OOH is the spin adduct generated from the interaction of superoxide radical ·$O_{2}^{-}$ and DMPO. For this system the following reactions occur:

h^+^ (graphene)+ H_2_O → H^+^ + ·OH (3)

DMPO+DMSO + ·OH →CH_3_(OH)SO + ·DMPO-CH_3_ (4)

(5) $$e^{-}\;(\mathrm{ZnO})+\;O_{2}\;\to \;\cdot O_{2}^{-}$$

(6) $$\mathrm{DMPO}+\;O_{2}^{-}\;\to \;\cdot \mathrm{DMPO-OOH}$$

The photodegradation mechanism is a general one and is valid for all types of dyes with specific degradation pathways depending on the intermediates which may participate in the reaction scheme. The radical oxygen species are responsible for the degradation of dyes to carbon dioxide, water, and other degradation species.

### 3.3. Electrochemical Studies

Cyclic voltammetry is one of the most useful methods employed for the determination of the electrocatalytic activity of nanostructured materials, which provide information about the reversibility of the redox process, the charge-transfer resistance (R_ct_), and the apparent heterogeneous electron transfer rate constant (K_app_). 

**Figure 12:**
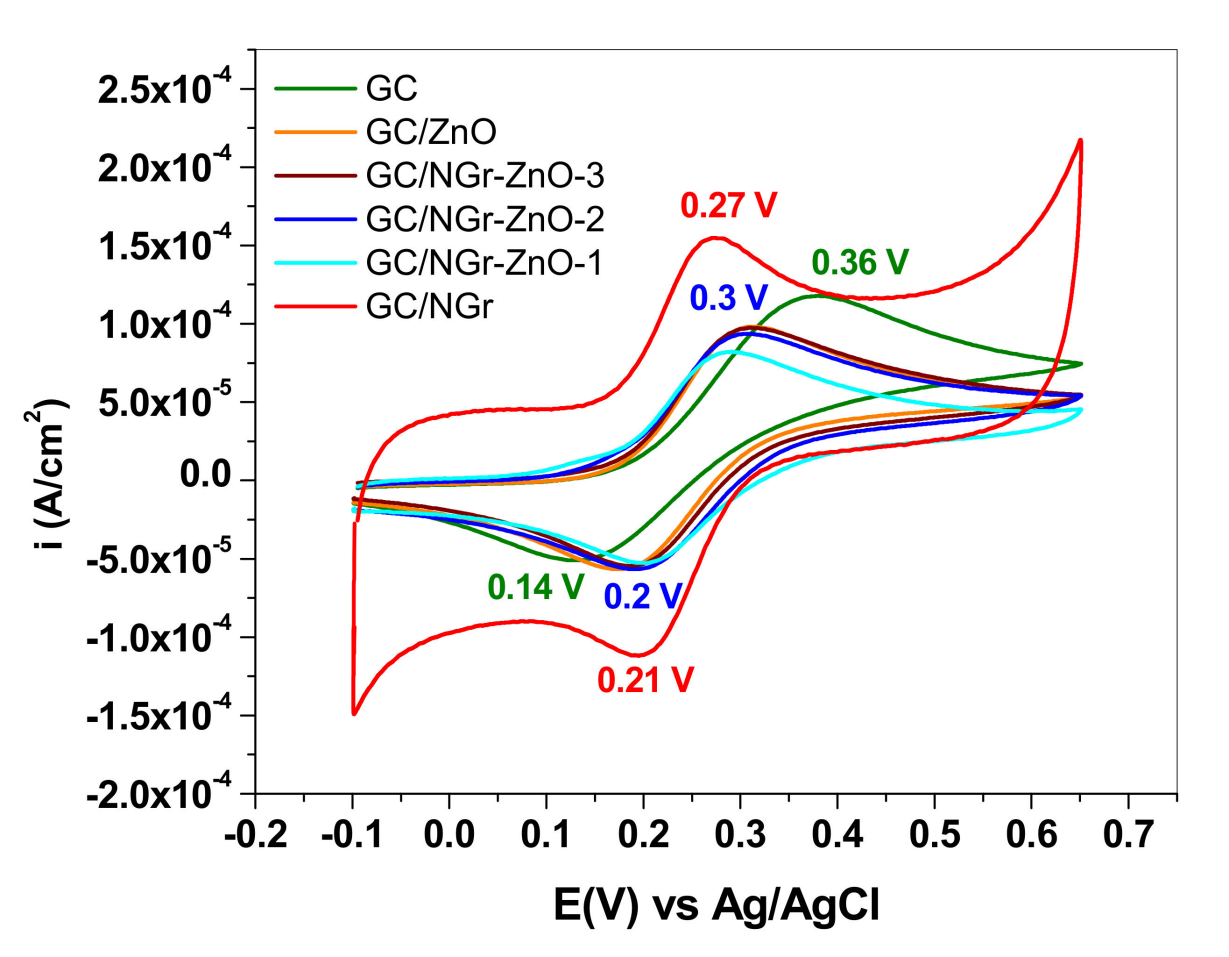
Cyclic voltammograms (current densities) recorded with bare glassy-carbon (GC) and GC-modified electrodes in 10^−3^ M K_4_[Fe(CN)_6_] (0.2 M KCl supporting electrolyte); scanning rate 10 mV/s.

Cyclic voltammograms were recorded with GC or GC-modified electrodes in solution containing 10^−3^ M K_4_[Fe(CN)_6_] (in 0.2 M KCl supporting electrolyte) at various scan rates (from 2 to 100 mV/s). Next, the active areas of GC and GC-modified electrodes were determined [48] and the values are listed in Table 5 along with other important parameters derived from cyclic voltammograms (Figure 12). The smallest active area was that corresponding to bare GC (0.028 cm^2^) and the largest to GC/NGr-ZnO-1 (0.0497 cm^2^). The other modified electrodes also had larger active areas, varying from 0.0313 to 0.0365 cm^2^.

The best CV parameters are those of the electrode modified with N-doped graphene (GC/NGr) which reflects the characteristics of a reversible redox process: I_pa_/I_pc_ ~ 1 and ΔE_p_ ~ 60 mV/n. In contrast, for all GC/NGr-ZnO modified electrodes, the parameters are considerably altered: I_pa_/I_pc_ > 1 with larger peak potential separation, from 83 to 115 mV. This can be related to the gradual increase of ZnO concentration within the composite materials. For bare GC electrode, the peak potential separation exceeds 200 mV and the anodic/cathodic waves are very broad, indicating a quasi-reversible redox process. 

In the case of GC/ZnO and GC/NGr-ZnO electrodes, the ZnO nanoparticles strongly influence the interaction of the surface with the redox species, due to the fact that ZnO surface charge depends on the solution pH. ZnO nanoparticles (isoelectric point of 9–10) [49] typically have hydroxyl groups attached to their surface [50]. At high pH, the chemisorbed protons (H^+^) may leave the particle surface generating a negatively-charged surface with partially bonded oxygen atoms (ZnO^−^). In contrast, at low pH the transfer of protons from the environment to the particle surface occurs, leading to a positively charged surface (ZnOH_2_^+^). 

In our case the solution pH was 6, therefore, the ZnO nanoparticles had a positive surface charge that attracted the negatively-charged ferrocyanide ions. Although ZnO nanoparticles had a beneficial effect in terms of surface coverage, their semiconducting properties led to the increase of the charge-transfer resistance (R_ct_) at the solution/electrode interface, as shown next by electrochemical impedance spectroscopy. The EIS spectra were recorded in 10^−3^ M K_4_[Fe(CN)_6_] (0.2 M KCl supporting electrolyte) within 0.1–10^6^ Hz frequency range.

**Figure 13:**
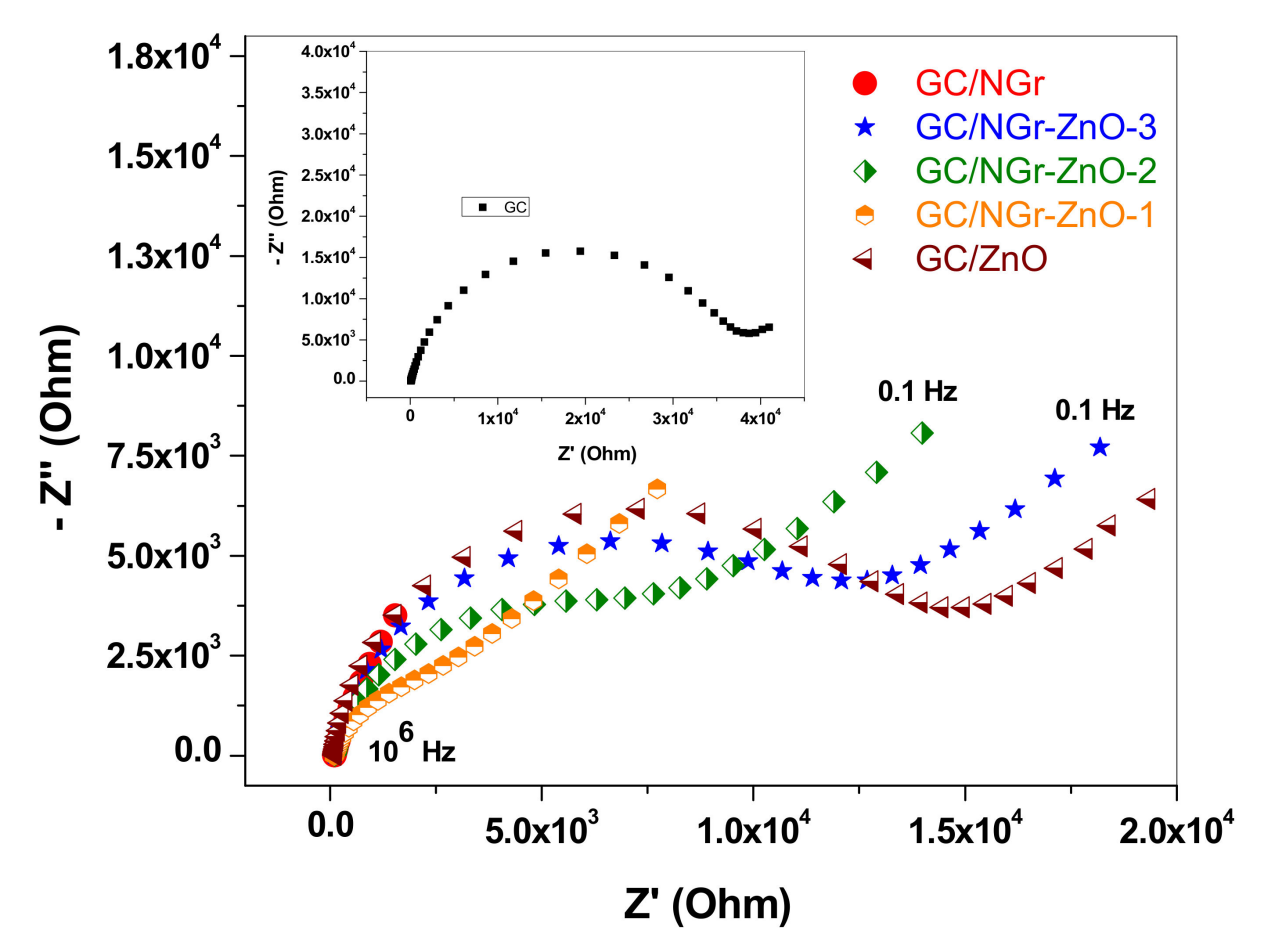
Nyquist plots obtained for bare GC (inset) and for the modified electrodes: GC/NGr (red), GC/NGr-ZnO-3 (blue), GC/NGr-ZnO-2 (green), GC/NGr-ZnO-1 (orange), and GC/ZnO (brown), in 10^−3^ M K_4_[Fe(CN)_6_] (0.2 M KCl supporting electrolyte); 0.1–10^6^ Hz frequency range; the spectra were recorded at the formal potential of each electrode.

Figure 13 shows the Nyquist plots for bare GC (inset) and for the modified electrodes: GC/NGr (red), GC/NGr-ZnO-1 (orange), GC/NGr-ZnO-2 (green), GC/NGr-ZnO-3 (blue), and GC/ZnO (brown). All electrodes exhibit a semicircle in the high-medium frequencies which appear due to the charge-transfer resistance (R_ct_) and a straight line at low frequencies, due to the diffusion of the redox specie towards the electrode surface. In the case of GC/NGr electrode the impedance values are significantly smaller (one order of magnitude) both for the semicircle and the diffusive line. 

**Figure 14:**
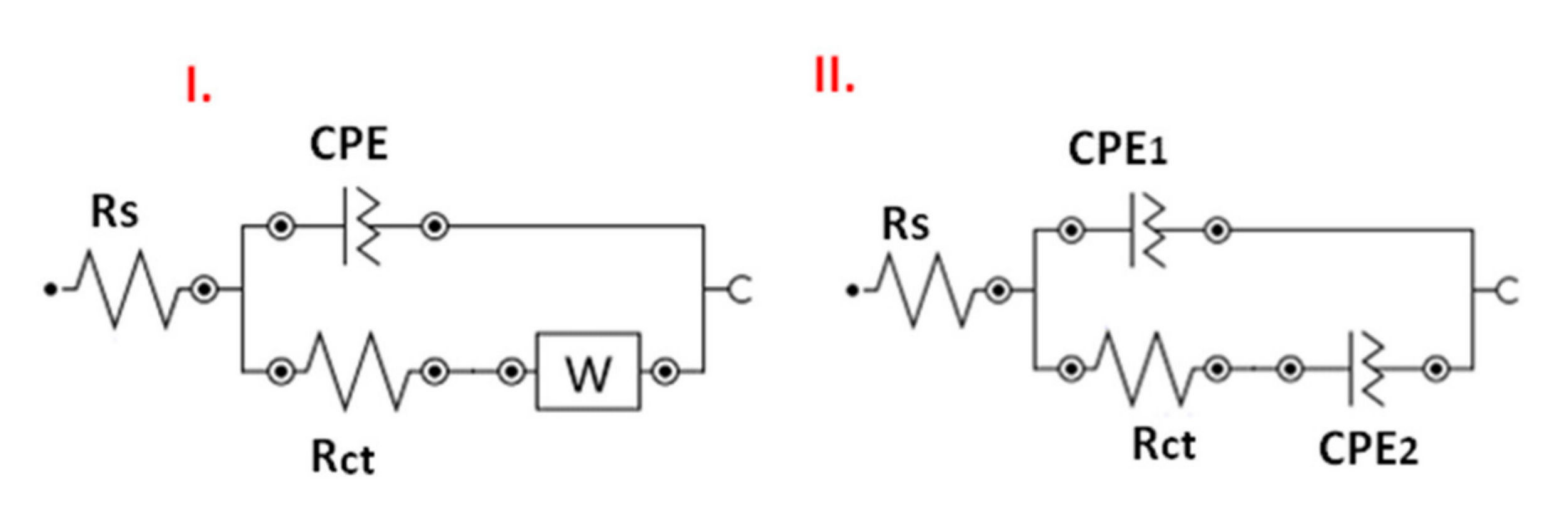
Modified Randles electrical equivalent circuits employed for fitting the experimental electrochemical impedance spectroscopy (EIS) data; circuit I was employed for GC, GC/ZnO, GC/NGr-ZnO-1, GC/NGr-ZnO-2, and GC/NGr-ZnO-3 electrodes; circuit II was employed for GC/NGr electrode.

Two modified Randles circuits were employed for fitting the experimental EIS data (see Figure 14).

The first circuit (I) contains the solution resistance (R_s_), the charge-transfer resistance (R_ct_) which reflects the easiness of electron transfer at the electrode/solution interface, the Warburg impedance (Z_W_) due to the diffusion of ions towards the interface, and a constant phase element (CPE) that models a rough surface (non-ideal capacitor). Such a circuit was employed to model the GC electrode as well as the electrodes containing ZnO nanoparticles: GC/ZnO, GC/NGr-ZnO-1, GC/NGr-ZnO-2, and GC/NGr-ZnO-3.

For the electrode modified only with N-doped graphene (GC/NGr) the interface was different than that of bare GC or modified with composites containing ZnO. The main reasons are related to the more porous structure formed by the successively deposited N-doped graphene layers. In this particular case, the best fit was obtained when the Warburg impedance was replaced with another CPE, while the other circuit elements were preserved (R_s_, R_ct_, and CPE_1_; Figure 14).

The values of the charge-transfer resistances were determined for all electrodes and can be seen in Table 6. The lowest value was that corresponding to GC/NGr electrode (6.01 Ω) while the largest was obtained for bare GC (36.8 kΩ). This is in excellent agreement with the previous CV measurements, which revealed that in the case of GC/NGr electrode the redox reaction had the characteristics of a reversible process and a quasi-reversible process in the case of bare GC.

For the other modified electrodes, the R_ct_ value was dependent on the amount of ZnO nanoparticles mixed with graphene and increased in the following order: GC/NGr-ZnO-1 (6.56 Ω), GC/NGr-ZnO-2 (7.91 kΩ), GC/NGr-ZnO-3 (11.6 kΩ),w and finally GC/ZnO (13.1 kΩ).

Using the R_ct_ value obtained for each electrode, the apparent heterogeneous electron transfer rate constant (K_app_) was calculated (Equation (7)) [51] and the values are listed in Table 6.

(7) $$K_{\mathrm{app}}\;=\;\frac{\mathrm{RT}}{n^{2}F^{2}AR_{\mathrm{ct}}C}$$

where R is the ideal gas constant (8.314 Joule/(mol·K)); T is the temperature (298 K); F is the Faraday constant (96485 C/mol); n is the number of electrons transferred during the redox reaction (n = 1); A is the active area of the electrode (cm^2^); R_ct_ is the charge-transfer resistance (Ω); C is the concentration of the redox specie (mol/cm^3^). 

The largest K_app_ value was obtained for NGr/GC electrode (1.01 cm/s) while the lowest was for bare GC (2.56 × 10^−4^ cm/s). For the other electrodes, the value is also dependent on the amount of ZnO nanoparticles present in the composite material, increasing in the following order: GC/NGr-ZnO-3 < GC/NGr-ZnO-2 < GC/NGr-ZnO-1. GC/NGr-ZnO-3 is a particular case due to the fact that the composite material contains a large quantity of ZnO. Therefore, the conductive properties of the composite materials are considerably altered and so R_ct_ and K_app_ values are close to those of GC/ZnO electrode.

## 4. Conclusions

Here we report a simple route to synthesize N-doped graphene-ZnO hybrid materials through the electrochemical exfoliation of graphite rods followed by a sol-gel process. UV–Vis spectroscopy was used to calculate the optical band gap of NGr, ZnO, and NGr-ZnO hybrid materials while electron paramagnetic resonance (EPR) coupled with the spin trapping probe technique evidenced the ROS generation process. XPS investigations showed that the amount of nitrogen is higher within the hybrid materials than in the stand-alone graphene flakes. Graphene structural defects increased with the ZnO content as seen by the increased number of defects (sp^3^) within its structure. The oxygen peak intensities corresponding to C-O, C=O, and COOH groups also increased with the increase of the ZnO content. Due to the electron transfer process from the metal atoms to graphene flakes, an additional “*n*” type doping appears in graphene while the ZnO nanocrystallites become “*p*” doped. This additional doping caused by electrons transferred from ZnO into N-doped graphene leads to a narrowing of the band gap within the hybrid material. A downward shift of the ZnO Fermi levels and energy bands was observed. It was attributed to “*p*” type doping which produced an inverse Burstein–Moss effect. The energy band diagram was constructed from UPS combined with UV–Vis data. All the samples have photocatalytic activity against rhodamine B solution, but the best performance was achieved with NGr-ZnO-3 sample, after 3 h of irradiation. The mechanism of photocatalysis concludes that ·OH species are generated mostly at the N-doped graphene–liquid interface while ·$O_{2}^{-}$ appears on the surface of ZnO nanocrystallites. The electrocatalytic performances of glassy-carbon electrodes modified with the hybrid materials were investigated and compared with those of pure ZnO and N-doped graphene.

## Figures and Tables

**Table 1:** Crystallite mean size and strain for the ZnO phase in the composite samples.

Sample	ZnO	NGr-ZnO-1	NGr-ZnO-2	NGr-ZnO-3
<*D*> (Å)	348	170	154	146
ε (%)	0.0011	0.0021	0.0023	0.0025

**Table 2:** The adsorption rate, photocatalytic activity, and apparent kinetic constant (*k_i_*) for the synthesized samples.

Sample	Adsorption Rate (%)	Photocatalytic Activity (%)	*k_i_* (min^−1^)	R^2^
ZnO	9	64	0.0052	0.99974
NGr-ZnO-1	36	96	0.0154	0.98828
NGr-ZnO-2	23	97	0.0184	0.92686
NGr-ZnO-3	26	100	0.0337	0.95355

**Table 3:** Comparison of photocatalytic performance of ZnO-rGO against different pollutants with previous reported literature.

Type of Irradiation	Photocatalyst Concentration	Degradation Rate/Time Irradiation	Pollutant	References
Visible	1.25 g/L	60%/60 min	rhodamine B (RhB)	[38]
Visible	1 g/L	95%/100 min	methylene blue	[39]
UV	1 g/L	100%/300 min	ofloxacin	[40]
UV	1 g/L	93%/60 min	methylene blue	[41]
UV	1.5 g /L	88%/260 min	methylene blue	[42]
Visible	1 g/L	92.9%/150 min	rhodamine B (RhB)	[43]
UV	0.5 g/L	100%/180 min	rhodamine B (RhB)	this work

**Table 4:** The doping degree and the values of different energies involved in the bands’ alignment. The N concentration was determined from XPS, the ionization energy E_i_ was measured with respect to the vacuum while VBM^ZnO^ values were given with respect to the Fermi energy (Highest occupied molecular orbital HOMO level). Band gaps were determined from UV–Vis spectra (Tauc plots). VBM: valence band maximum; UPS: UV photoelectron spectroscopy.

Sample	N (at.%) XPS	E_i_ (eV) UPS	VBM (eV) UPS	E_g_^NGr^ (eV)	E_g_^ZnO^ (eV) UV–Vis
NGr-ZnO-1	7.7	−4.66	−3.14	0.7	3.15
NGr-ZnO-2	7.5	−4.64	−3.38	0.6	3.18
NGr-ZnO-3	18.6	−4.63	−3.55	<0.1	3.26
ZnO	−	−4.78	−2.49	−	3.32
NGr	3.8	−5.06	−0.57	~0.5	−

**Table 5:** The electrochemical parameters of bare and modified electrodes.

Electrode	ΔE_p_ mV/n	I_pa_ µA	I_pc_ µA	I_pa_/I_pc_	Q_a_ µC	A cm^2^
GC/NGr	60	2.86	−2.81	1.02	0.379	0.0365
GC/NGr-ZnO-1	83	3.09	2.78	1.11	0.644	0.0497
GC/NGr-ZnO-2	98	2.45	−2.25	1.08	0.554	0.0343
GC/NGr-ZnO-3	115	2.67	−2.43	1.099	0.608	0.0354
GC/ZnO	125	2.46	−2.24	1.099	0.550	0.0313
GC	220	2.32	−1.85	1.25	0.547	0.028

ΔE_p_—peak potential separation; I_pa,c_—anodic/cathodic peak current; Q_a_—anodic peak area; A—active area.

**Table 6:** The values of R_ct_ and K_app_ for bare and GC-modified electrodes.

Electrode	GC/ NGr	GC/ NGr-ZnO-1	GC/ NGr-ZnO-2	GC/ NGr-ZnO-3	GC/ ZnO	GC
R_ct_ (Ω)	6.01	6.56	7910	11,600	13,100	36,800
K_app_ (cm/s)	1.01	8.9 × 10^−1^	9.75 × 10^−4^	6.44 × 10^−4^	6.45 × 10^−4^	2.56 × 10^−4^

## References

[1] Smazna D., Shree S., Polonsky O., Lamaka S., Baum M., Zheludkevich M., Faupel F., Adelung R., Mishra Y.K. (2019). Mutual interplay of ZnO micro- and nanowires and methylene blue during cyclic photocatalysis. J. Environ. Chem. Eng..

[2] Toloman D., Mesaros A., Popa A., Silipas T.D., Neamtu S., Katona G. (2016). V-doped ZnO particles: Synthesis, structural, optical and photocatalytic properties. J. Mater. Sci. Mater. Electron..

[3] Qi K., Cheng B., Yu J., Ho W. (2017). Review on the improvement of the photocatalytic and antibacterial activities of ZnO. J. Alloys Compd..

[4] Sa-nguanprang S., Phuruangrat A., Thongtem T., Thongtem S. (2020). Characterization and photocatalysis of visible-light-driven Dy-doped ZnO nanoparticles synthesized by tartaric acid-assisted combustion method. Inorg. Chem. Commun..

[5] Velázquez-Nevárez G.A., Vargas-García J.R., Aguilar-Hernández J., Vega-Becerra O.E., Chen F., Shen Q., Zhang L. (2016). Optical and electrical properties of (002)-oriented ZnO films prepared on amorphous substrates by sol-gel spin-coating. Mat. Res..

[6] Moqbel R.A., Gondal M.A., Qahtan T.F., Dastageer M.A. (2018). Pulsed laser synthesis in liquid of efficient visible-light-active ZnO/rGO nanocomposites for improved photo-catalytic activity. Mater. Res. Express..

[7] Jayachandiran J., Yesuraj J., Arivanandhan M., Raja A., Suthanthiraraj S.A., Jayavel R., Nedumaran D. (2018). Synthesis and electrochemical studies of rGO/ZnO nanocomposite for supercapacitor application. J. Inorg. Organomet. Polym. Mater..

[8] Brisebois P.P., Siaj M. (2020). Harvesting graphene oxide-years 1859 to 2019: A review of its structure, synthesis, properties and exfoliation. J. Mater. Chem. C..

[9] Tarcan R., Todor-Boer O., Petrovai I., Leordean C., Astilean S., Botiz I. (2020). Reduced graphene oxide today. J. Mater. Chem. C..

[10] Li B., Liu T., Wang Y., Wang Z. (2012). ZnO/graphene-oxide nanocomposite with remarkably enhanced visible-light-driven photocatalytic performance. J. Colloid Interface Sci..

[11] Jabeen M., Ishaq M., Song W., Xu L., Maqsood I., Deng Q. (2017). UV-Assisted Photocatalytic Synthesis of ZnO-reduced graphene oxide nanocomposites with enhanced photocatalytic performance in degradation of Methylene Blue. ECS J. Solid State Sci. Technol..

[12] Alam N.S., Sharma N., Kumar L. (2017). Synthesis of graphene oxide (GO) by modified hummers method and its thermal reduction to obtain reduced graphene oxide (rGO)*. Graphene.

[13] Parul K.K., Badru R., Singh P.P., Kaushal S. (2020). Photodegradation of organic pollutants using heterojunctions: A review. J. Environ. Chem. Eng..

[14] Liu F., Wang C., Sui X., Riaz M.A., Xu M., Wei L., Chen Y. (2019). Synthesis of graphene materials by electrochemical exfoliation: Recent progress and future potential. Carbon Energy.

[15] Pogacean F., Coros M., Magerusan L., Mirel V., Turza A., Katona G., Stefan-van Staden R.I., Pruneanu S. (2019). Exfoliation of graphite rods via pulses of current for graphene synthesis: Sensitive detection of 8-hydroxy-2′-deoxyguanosine. Talanta.

[16] Kindalkar V.S., Sandeep K.M., Kumara K., Dharmaprakash S.M. (2019). Sol-gel synthesized spin coated GO: ZnO composite thin films: Optical, structural and electrical studies. Mater. Res. Express.

[17] Chu H.O., Wang Q., Shi Y.J., Song S.G., Liu W.G., Zhou S., Gibson D., Alajlani Y., Li C. (2020). Structural, optical properties and optical modelling of hydrothermal chemical growth derived ZnO nanowires. Trans. Nonferrous Met. Soc. China (Engl. Ed.).

[18] Liu X., Pan L., Lv T., Lu T., Zhu G., Sun Z., Sun C. (2011). Microwave-assisted synthesis of ZnO-graphene composite for photocatalytic reduction of Cr(VI). Catal. Sci. Technol..

[19] Jana A., Gregory D.H. (2020). Microwave-assisted synthesis of ZnO-rGO core-shell nanorod hybrids with photo- and electro-catalytic activity. Chem. Eur. J..

[20] Zhang W., Yang Y., Ziemann E., Be’Er A., Bashouti M.Y., Elimelech M., Bernstein R. (2019). One-step sonochemical synthesis of a reduced graphene oxide-ZnO nanocomposite with antibacterial and antibiofouling properties. Environ. Sci. Nano.

[21] Louis J., Kavitha M.K., Anjana V., Jayaraj M., John H. (2020). A facile surfactant assisted hydrothermal synthesis of ZnO and graphene loaded ZnO for efficient photocatalytic self-cleaning. Mater. Res. Express.

[22] Lonkar S.P., Pillai V., Abdala A. (2019). Solvent-free synthesis of ZnO-graphene nanocomposite with superior photocatalytic activity. Appl. Surf. Sci..

[23] Wang L., Li Z., Chen J., Huang Y., Zhang H., Qiu H. (2019). Enhanced photocatalytic degradation of methyl orange by porous graphene/ZnO nanocomposite. Environ. Pollut..

[24] Ramanathan S., Selvin S.P., Obadiah A., Durairaj A., Santhoshkumar P., Lydia S., Ramasundaram S., Vasanthkumar S. (2019). Synthesis of reduced graphene oxide/ZnO nanocomposites using grape fruit extract and Eichhornia crassipes leaf extract and a comparative study of their photocatalytic property in degrading Rhodamine B dye. J. Environ. Heal. Sci. Eng..

[25] Anandkumar J., Mandal B. (2011). Adsorption of chromium(VI) and Rhodamine B by surface modified tannery waste: Kinetic, mechanistic and thermodynamic studies. J. Hazard. Mater..

[26] Umar M., Aziz H.A., Rashed N.M. (2013). Photocatalytic degradation of organic pollutants in water. Organic Pollutants—Monitoring, Risk and Treatment.

[27] Zhang Y.-N., Niu Q., Gu X., Yang N., Zhao G. (2019). Recent progress on carbon nanomaterials for the electrochemical detection and removal of environmental pollutants. Nanoscale.

[28] Król A., Pomastowski P., Rafińska K., Railean-Plugaru V., Buszewski B. (2017). Zinc oxide nanoparticles: Synthesis, antiseptic activity and toxicity mechanism. Adv. Colloid. Interface Sci..

[29] Zhong L., Liu H., Samal M., Yun K. (2018). Synthesis of ZnO nanoparticles-decorated spindle-shaped graphene oxide for application in synergistic antibacterial activity. J. Photochem. Photobiol. B Boil..

[30] Cumpson P.J., Seah M.P. (1997). Elastic scattering corrections in AES and XPS II. Estimating attenuation lengths and conditions required for their valid use in overlayer/substrate experiments. Surf. Interface Anal..

[31] Garcia-Martinez O., Rojas R., Vila E., Devidales J. (1993). Microstructural characterization of nanocrystals of ZnO and CuO obtained from basic salts. Solid State Ionics.

[32] Kraus W., Nolzeb G. (1996). POWDER CELL—A program for the representation and manipulation of crystal structures and calculation of the resulting X-ray powder patterns. J. Appl. Crystallogr..

[33] Hao C., Yang Y., Shen Y., Feng F., Wang X., Zhao Y., Ge C. (2016). Liquid phase-based ultrasonic-assisted synthesis of G–ZnO nanocomposites and its sunlight photocatalytic activity. Mater. Des..

[34] Nguyen V.Q., Baynosa M.L., Nguyen V.H., Tuma D., Lee Y.R., Shima J.-J. (2019). Solvent-driven morphology-controlled synthesis of highly efficient long-life ZnO/graphene nanocomposite photocatalysts for the practical degradation of organic wastewater under solar light. Appl. Surf. Sci..

[35] Tang L., Ji R., Li X., Teng K.S., Lau S.P. (2013). Energy-level structure of nitrogen-doped graphene quantum dots. J. Mater. Chem. C.

[36] Deng H., Yao L., Huang Q.-A., Su Q., Zhang J., Zhang F., Du G. (2017). Facile assembly of a S@carbon nanotubes/polyaniline/graphene composite for lithium–sulfur batteries. RSC Adv..

[37] Usachov D., Vilkov O., Grüneis A., Häberer D., Fedorov A., Adamchuk V.K., Preobrajenski A.B., Dudin P., Barinov A., Oehzelt M. (2011). Nitrogen-Doped Graphene: Efficient Growth, Structure, and Electronic Properties. Nano Lett..

[38] Neena D., Kondamareddy K.K., Humayun M., Mohan V.B., Lu D., Fu D., Gao W. (2019). Fabrication of ZnO/N-rGO composite as highly efficient visible-light photocatalyst for 2,4-DCP degradation and H_2_ evolution. Appl. Surf. Sci..

[39] Wang C.-C., Shieu F.-S., Shih H.C. (2020). Enhanced photodegradation by RGO/ZnO core-shell nanostructures. J. Environ. Chem. Eng..

[40] Sharma P., Kumar N., Chauhan R., Singh V., Srivastava V.C., Bhatnagar R. (2020). Growth of hierarchical ZnO nano flower on large functionalized rGO sheet for superior photocatalytic mineralization of antibiotic. Chem. Eng. J..

[41] Jiang H., Zhang X., Gu W., Feng X., Zhang L., Weng Y. (2018). Synthesis of ZnO particles with multi-layer and biomorphic porous microstructures and ZnO/rGO composites and their applications for photocatalysis. Chem. Phys. Lett..

[42] Lv T., Pan L., Liu X., Sun Z. (2012). Enhanced photocatalytic degradation of methylene blue by ZnO–reduced graphene oxide–carbon nanotube composites synthesized via microwave-assisted reaction. Catal. Sci. Technol..

[43] Li X., Wang Q., Zhao Y., Wu W., Chen J., Meng H. (2013). Green synthesis and photo-catalytic performances for ZnO-reduced graphene oxide nanocomposites. J. Colloid Interface Sci..

[44] Pastrana-Martínez L.M., Morales-Torres S., Likodimos V., Figueiredo J.L., Faria J.L., Falaras P., Silva A.M. (2012). Advanced nanostructured photocatalysts based on reduced graphene oxide–TiO_2_ composites for degradation of diphenhydramine pharmaceutical and methyl orange dye. Appl. Catal. B Environ..

[45] Williams G., Seger B., Kamat P.V. (2008). TiO_2_-Graphene Nanocomposites. UV-Assisted Photocatalytic Reduction of Graphene Oxide. ACS Nano.

[46] Klein S.M., Cohen G., Cederbaum A.I. (1981). Production of formaldehyde during metabolism of dimethyl sulfoxide by hydroxyl radical-generating systems. Biochemistry.

[47] Sushkov D., Gritsan N., Weiner L. (1987). Generation of OH radical during enzymatic reduction of 9,10-anthraquinone-2-sulphonate Can semiquinone decompose hydrogen peroxide?. FEBS Lett..

[48] Ferrari A.G.-M., Foster C.W., Kelly P.J., Brownson D.A.C., Banks C.E. (2018). Determination of the Electrochemical Area of Screen-Printed Electrochemical Sensing Platforms. Biosensensors.

[49] Degen A., Kosec M. (2000). Effect of pH and impurities on the surface charge of zinc oxide in aqueous solution. J. Eur. Ceram. Soc..

[50] Qu F., Morais P.C. (1999). Energy levels in metal oxide semiconductor quantum dots in water-based colloids. J. Chem. Phys..

[51] Nkosi D., Pillay J., Ozoemena K.I., Nouneh K., Oyama M. (2010). Heterogeneous electron transfer kinetics and electrocatalytic behaviour of mixed self-assembled ferrocenes and SWCNT layers. Phys. Chem. Chem. Phys..

